# Defining the cultured and uncultured bacterial fractions in *Cannabis* seeds

**DOI:** 10.1186/s40793-025-00731-4

**Published:** 2025-06-11

**Authors:** Carolina Lobato, Ahmed Abdelfattah, Gabriele Berg, Tomislav Cernava

**Affiliations:** 1 Institute of Environmental Biotechnology, Graz University of Technology, Petersgasse 12, Graz, 8010 Austria; 2 Leibniz Institute for Agricultural Engineering and Bioeconomy, Max-Eyth-Allee 100, Potsdam, 14 469 Germany; 3 Institute for Biochemistry and Biology, University of Potsdam, Karl-Liebknecht-Str. 24-25, Potsdam OT Golm, 14 476 Germany; 4 School of Biological Sciences, Faculty of Environmental and Life Sciences, Highfield Campus, Southampton, SO17 1BJ UK

**Keywords:** Bacteria, Cannabis, Co-occurrence network, Culturing, Microbial dark matter, Microbial interactions, Microbiome breeding, Plant fitness, Plant microbiome, Seed endophytes

## Abstract

**Background:**

Seeds provide a unique environment shaped by co-evolutionary processes, hosting diverse microbial communities. While microbiome studies have uncovered an extensive diversity of microorganisms, culture-based approaches remain crucial for understanding microbial potential and functional interactions. However, the factors influencing microbial culturability within seeds are not well understood.

**Results:**

In this study, we investigated the culturing patterns of bacteria inside *Cannabis* seeds, assessing their phylogenetic diversity, abundance, and putative interactions. Bacteria were cultured from 54 different *Cannabis* accessions using germinated seeds and a range of nutrient media including those supplemented with *Cannabis* extracts. The cultured fraction consisted of taxa from five prominent classes—*Gammaproteobacteria*, *Bacilli*, *Actinobacteria*, *Alphaproteobacteria*, and *Bacteroidia*—encompassing 36 genera. Despite representing only 6.3% of the total microbiota, these cultured bacteria accounted for 89.2% of the microbial population. Almost 60% of the amplicon sequence variants (ASVs) were phylogenetically distant from cultured taxa. Rare bacterial groups such as *Acidobacteriae* and *Verrucomicrobiae*, known for their plant growth-promoting traits, were exclusively found in the uncultured fraction. Network analyses revealed that uncultured taxa are centralized and more connected to hubs, suggesting that interspecies interactions strongly influence culturability.

**Conclusion:**

Our findings highlight the limitations of culture-based methods in capturing the full microbial diversity of *Cannabis* seeds and emphasize the importance of microbial interactions in determining culturability. The strong network connectivity of uncultured taxa suggests that interdependencies and competition within the seed microbiome may hinder the isolation of key bacterial groups. These insights provide a framework for refining cultivation strategies to recover ecologically significant microbes with potential agricultural applications.

**Supplementary Information:**

The online version contains supplementary material available at 10.1186/s40793-025-00731-4.

## Background

Culture-independent approaches have expanded our knowledge of microbial life, revealing an extraordinary diversity of microorganisms and allowing to glimpse into their ecological roles [1, 2]. However, culturing of microorganisms remains crucial for exploring microbial functionality and interactions as well as for refining -omics databases by facilitating high-resolution genomic analyses [3]. For instance, the direct observation of physiological traits with live microbial cultures can offer deeper insights into regulatory mechanisms and metabolic pathways [4]. However, substantial fractions of microbial life remain elusive to culturing across environments [5, 6], representing untapped reservoirs of genetic and functional diversity with significant implications for ecosystem processes [2]. While uncultured microbial fractions have been extensively reported in soils and other major biomes [6, 7], studies addressing these fractions for plant compartments remain scarce. In the rhizosphere, uncultured fractions are dominated by oligotrophic taxa with key roles in nutrient cycling and plant growth promotion, such as *Acidobacteria*, *Verrucomicrobia*, *Planctomycetes*, and *Gemmatimonadetes* [8]. Among studies in the phyllosphere, uncultured taxa such as *Bacteroidales*, *Enterobacteriales*, *Myxococales* and *Sphingobacteriales* have been identified [9], but the uncultured fractions of seeds remain largely unexplored [10].

Seeds serve as vectors for microbial transmission, perpetuating microorganisms across plant generations [11]. Endophytic microbes can be transmitted to seeds through the plant’s vascular movement, or reproductive structures [12]. Additional microbial contributions through horizontal transmission can also occur during developmental and post-developmental stages; however, the relative importance of these pathways for the seed microbiome remains an area of active research [12–15]. Seed-borne microbes can contribute to the development and resilience of the new plant by accomplishing important functions such as nitrogen fixation (supporting plant nitrogen uptake), phytohormone modulation (influencing release from dormancy and germination), nutrient solubilization (enhancing, for example, phosphorus and potassium availability), and siderophore production (facilitating iron acquisition and pathogen suppression) [16]. A considerable number of studies has provided deeper insights into the seed microbiome diversity and functions across many plant species, using both culture-dependent and -independent approaches [17–27], including in *Cannabis* [28–30]. Despite these insights, few studies have specifically addressed challenges [31] or strategies [32, 33] for improving the culturing of seed-associated microorganisms.

Seed endophytic communities have relatively low diversity and cell density compared to microbial communities in other plant compartments [34]. To persist under resource-limiting and selective seed conditions, and maintain stable seed microbial communities, these microbes may have specific nutrient requirements, reduced growth rates [35], or enter viable but non-culturable (VBNC) states [36, 37] that hinder microbial recovery in culture [38–40]. Moreover, the constrained seed environment is likely to amplify microbe-microbe interactions [41], including the exchange of genetic and molecular signals mediating complex interspecies dynamics, such as the production of secondary metabolites, siderophores and quorum sensing molecules [42]. Despite this, the role of microbial interactions in shaping the culturability of seed endophytes has been largely overlooked. Here, we cultured *Cannabis* seed endophytic bacteria using different standardized and tailored media with *Cannabis* extracts, to target bacteria with different metabolic preferences, as well as different dilutions, and prolonged incubation times that aimed to mitigate biases toward fast-growing taxa [3]. Additionally, seed soaking and germination were used to activate dormant microbes and provide a more comprehensive view of the *Cannabis* seed endophytic communities [43]. As a reference for investigating cultured and uncultured fractions of bacteria, as well as potential factors influencing culturability, we used the seed bacterial community dataset from our previous study, including 46 *Cannabis* genotypes, with community profiles obtained under the same treatment and plant developmental stage as this study [28]. We hypothesized that a considerable fraction of the *Cannabis* seed microbiome remains uncultured, despite our extended approach to traditional culturing methods, in part due to microbial interactions within the seed environment. By shedding light on the uncultured fraction of seed microbial diversity, this study lays the groundwork for refining future cultivation strategies to recover missing bacterial taxa. Overall, we aim to uncover culturability patterns that explain why some seed bacterial taxa can be cultured while others remain elusive.

## Materials and methods

### Seed collection

In this study, we attempted to isolate bacterial constituents of the seed microbiota from 91 *Cannabis* seed accessions, originally collected from diverse geographic regions across the world but sourced from institutions and seed companies based in Europe. Fifty-four accessions were successfully implemented in the isolation approach, including 32 accessions previously described in Lobato *et al.* [28], while others only occur in this study (*n* = 22) (Fig. S1). Comprehensive details regarding all *Cannabis* accessions included in this study are provided in Table S1.

### Bacterial isolation and sequencing

Seeds underwent a pre-treatment process to eliminate non-endophytic microorganisms. Initially, seeds were soaked in sterile deionized water for 4 h on a shaker at 125 rpm. Subsequently, surface sterilization was conducted using a 4% solution of sodium hypochlorite (NaClO) for 5 min with agitation, followed by three 5-min rinses in sterile water. Seed sterilization was confirmed by inoculating 100 µL of the final rinse water onto Nutrient Agar II (NA II) plates. Seeds were then germinated under sterile conditions until the emergence of radicle and cotyledons. A broad spectrum of nutrients was used for bacterial isolation including minimal and diluted media amended with 1% (v/v) *Cannabis* extracts aimed at slow-growing and *Cannabis*-specific bacteria (Table S2). *Cannabis* extracts were prepared by blending the tissues of juvenile plants with 10 mL of water per gram of tissue, followed by centrifugation at 7000 g for 15 min and sterilization of the supernatant with a 0.4 μm followed by a 0.2 μm filter into pre-autoclaved media to preserve the integrity of its components. Tissue solutions for bacterial isolation were prepared by crushing three seedlings of the same genotype with 4 mL of 0.85% NaCl and used for plating in three technical replicates per media at 10^0^ to 10^− 4^ dilutions. Bacterial cultures were incubated at room temperature (25 °C) in the dark until colony formation, with incubation periods extending up to 30 days, beyond which media degradation (e.g., drying and loss of integrity) limited further culturing. Pure bacterial cultures were obtained based on dissimilar appearance of colonies (i.e., shape, size, edge, chromogenesis, opacity, elevation, surface and consistency) that were present in at least two technical replicates to select the maximum of diversity and exclude possible contaminants.

Bacteria obtained from the same *Cannabis* genotype were pooled and DNA extraction was performed using the MasterPure Complete DNA and RNA Purification Kit (Epicentre, Madison, WI, USA), following the manufacturer’s instructions. Additional steps were included for enhanced lysis efficiency, i.e., incorporation of lysozyme in the lysis buffer, and a mechanical lysis step using Screw Cap Micro Tubes (Thermo Fisher Scientific, DE, USA). These tubes contained 1.4 mm ceramic spheres, 0.1 mm silica spheres, and one 4 mm glass sphere. Sample were disrupted in a FastPrep-24 instrument (MP Biomedicals, CA, USA) at 6 m/s for 25 seconds, twice, and kept one minute on ice between repetitions. DNA integrity was verified by agarose gel electrophoresis and concentration was measured using a Nanodrop 2000 spectrophotometer (Thermo Fisher Scientific, DE, USA). The full-length 16S rRNA gene was amplified using the 27f (5’-AGRGTTYGATYMTGGCTCAG-3’) and 1492r (5’-RGYTACCTTGTTACGACTT-3’) universal primer set from the extracted DNA. Primers were tailed with sample-specific PacBio barcode sequences to allow for multiplexed sequencing (PacBio, CA, USA). PCR amplification, was performed with an initial denaturing step at 95 °C for 3 min, followed by 25 cycles of denaturing at 95 °C for 30 s, annealing at 57 °C for 30 s and extension at 72 °C for 60 s. PCR reactions were performed in a total volume of 25 µL and in three technical replicates using 2x KAPA HiFi HotStart Ready Mix (KAPA Biosystems, MA, USA), 0.3 µM of each primer, PCR-grade water, and 0.25 ng/µL of DNA template. Post-amplification quality control was performed using the Qubit dsDNA HS Assay Kit on a Qubit 4 Fluorometer (Thermo Fisher Scientific, DE, USA). Amplified DNA was pooled in equimolar concentration, including negative controls for PCR amplification, and PCR products amplified from the ZymoBIOMICS Microbial Community DNA Standard (Zymo Research, CA, USA) to infer bias and errors introduced by sequencing library preparation. SMRTbell libraries were sequenced on a PacBio Sequel platform with v3.0 chemistry (GENEWIZ GmbH, Germany). The generated subreads were demultiplexed and circular consensus sequencing (CCS) reads were obtained using the CCS algorithm within PacBio ccs v4.2.0 using the default parameters. A total of 334 637 CCS reads with a mean length of 1514 bases, a read score of 0.999 and a mean of 30 passes were obtained.

### Bioinformatic processing

#### Cultured bacterial seed endophytes

CCS reads were first demultiplexed using Lima v2.9.0 [44], specifying that different barcodes were attached at the ends of an insert using the flag *asymmetric*. DADA2 v1.32.0 was implemented in R v4.4.1 [45] to infer the amplicon sequence variants (ASVs) as proposed by Callahan *et al.* [46]. The standard processing steps in the DADA2 workflow include quality filtering, dereplication, learning the dataset-specific error model, ASV inference, chimera removal and taxonomic assignment with VSEARCH and the Silva v128 database [47, 48]. Detected contaminants were excluded from the dataset (Fig. S2). A total of 419 ASVs in 202 111 high-quality reads, were retained in 132 samples. The full-length 16S rRNA gene dataset was deposited in the European Nucleotide Archive (ENA; https://www.ebi.ac.uk/ena) under the accession number PRJEB83654.

#### Total bacterial seed community

Demultiplexed 16S rRNA gene pair-end reads from the V4 region were acquired from our previous study [28] that is publicly available (ENA; https://www.ebi.ac.uk/ena) under the accession number PRJEB64469, where the pre-processing was done following the workflow as described [28]. Briefly, DADA2 was employed within QIIME2 v2023.5 for read quality filtering, denoising, merging, and generating amplicon sequence variants (ASVs) and the feature Tables [49, 50]. Taxonomic classification was conducted using the VSEARCH in QIIME2 and the Silva v138 database for 16S rRNA gene sequences [47, 51]. Sequences identified as unassigned, non-target (e.g., chloroplasts, mitochondria, archaea), or of low quality were removed and samples with fewer than 1000 reads (*n* = 6) were excluded. Contaminant ASVs were identified and removed from the dataset using the decontam package [52] in R v4.4.1 [45] based on prevalence with the Fisher method. The final dataset included 36 996 902 high-quality reads (82 033 mean reads/sample) and 5297 bacterial ASVs across 46 distinct *Cannabis* accessions (Table S1).

### Bioinformatic analysis

The datasets derived from isolated, laboratory-grown bacterial endophytes and those representing the total bacterial community directly extracted from seeds are henceforth designated as the cultured and community datasets, respectively. The feature table and taxonomic information of both datasets were analyzed using phyloseq [53] and tidyverse [54] in R v4.4.1 [45] unless otherwise specified. The comparison between datasets was performed using NCBI BLAST + blastn with the megablast tool in Galaxy [55, 56], with ungapped alignment only and minimum query coverage on the 16S rRNA gene V4 region of 100% (Table S3). Community ASVs were classified based on the highest sequence identity percentage hit with the cultured dataset, and binned based on arbitrary thresholds to further explore phylogenetic distances between cultured and uncultured taxa. We considered a > 99% sequence identity threshold for the identification of the cultured fraction at species-level, and account for small variations between the two sequencing methods [57]. The remaining thresholds bin the uncultured fraction according to the upper 95% confidence interval (CI) of the median 16S rRNA gene identity for each taxonomic level as shown by Yarza *et al.* [58]. According to this, ASVs in the community dataset with sequence identity percentages to the isolates below 85.93% were classified as phylogenetically distant. Further, taxonomic assignments described in this study are according to the Silva v138 database from rRNA gene sequences, to ensure consistency with the taxonomy described in Lobato *et al.* [28]. The phylogenetic tree was generated with Mega11 v11.0.13 with 99 bootstrap replications and the nearest-neighbor interchange method after alignment with MUSCLE [59, 60] and rendered using the Interactive Tree Of Life tool (iTOL) v6 [61]. The network was generated with Flashweave v0.19.2 [62] in Julia v1.10.4 [63] on the community feature table, incorporating genotype-level metadata and the following parameters: sensitive = true, max_k = 3, normalize = true, heterogeneous = true, n_obs_min = 20, FDR = true, alpha = 0.05, time_limit = -1, conv = 0.001, feed_forward = true. FlashWeave addresses known sources of bias, including compositionality effects, shared-niche dependencies, and sequencing artifacts. Cytoscape v3.10.2 [64] was used for network visualization and calculation of the centrality measures, and visualization. We conducted hub classification, where nodes had to simultaneously fulfil the arbitrary threshold values for degree (> 7), betweenness (> 0.1) and closeness (> 0.3) centralities to describe their high connectivity and presence [65]. Network analysis was conducted using igraph in R [66].

## Results

More than 90% of the isolates matched with ASVs from the community dataset.

**Fig. 1:**
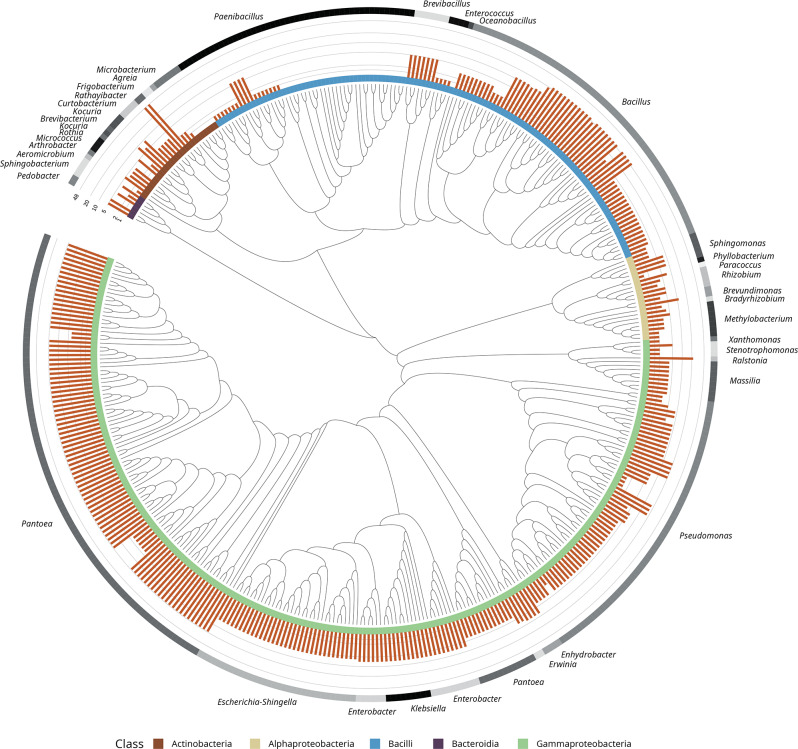
Cultured bacterial endophytic diversity in the *Cannabis* seed microbiome. The circular phylogenetic tree shows the 419 bacterial amplicon sequence variants (ASVs) identified through culturing. The inner color-coded stripes denote bacterial classes (e.g., *Actinobacteria*, *Alphaproteobacteria*, *Bacilli*, *Bacteroidia*, and *Gammaproteobacteria*), while the outer stripes annotate the corresponding genera. The orange bars quantify the number of community ASVs assigned to each isolate with > 99% sequence identity. This visualization highlights the taxonomy and phylogenetic distribution of the *Cannabis* seed-associated bacterial endophytes

Only 54 *Cannabis* accessions, out of the 91 tested in this study, yielded seed endophytic bacteria. From them, a total of 1192 bacterial pure cultures were obtained. The cultured taxa corresponded to 419 ASVs from 36 genera and 5 classes (Fig. 1). Most of the isolates belonged to *Gammaproteobacteria* (58%), followed by *Bacilli* (29.36%), *Actinobacteria* (6.2%), *Alphaproteobacteria* (5.01%), and *Bacteroidia* (1.43%). Despite that, the diversity of genera covered by *Gammaproteobacteria* and *Bacilli* (*n* = 11 and *n* = 5, respectively) was similar to those of *Actinobacteria* (*n* = 11) and *Alphaproteobacteria* (*n* = 7). Representatives of *Pantoea* contributed to 25.78% of the isolates, followed by *Bacillus* (14.79%), *Pseudomonas* (13.84%), and *Paenibacillus* (11.69%). Moreover, *Bacillus* and *Pantoea* were highly prevalent, being detected in 48.15% and 40.74% of the genotypes, respectively. (Fig. S3).

A high percentage of the isolates (90.45%) showed assignments at species level (> 99% sequence identity) with the community dataset; exceptions included representatives from *Actinobacteria*, such as *Rothia* (*n* = 1) and *Microbacterium* (*n* = 6), *Bacilli*, mostly *Paenibacillus* (*n* = 31), and *Bacteroidia* (*Sphingobacterium*, *n* = 1), but not *Gammaproteobacteria* nor *Alphaproteobacteria*. The highest number of assignments was found for the two representatives of *Rathayibacter* (*n* = 48), followed by *Bacillus* (*n* = 23) and *Ralstonia* (*n* = 20), while 33.25% of the isolates matched with less than 5 ASVs at 99% sequence identity in the community dataset. For many of the isolated genera (e.g., *Pantoea*, *Bacillus*, and *Pseudomonas*), representatives were assigned to the same community ASVs, revealing high sequence identity between isolates of the same genus (Table S3).

Only a small fraction of the total community ASVs were recovered but they cover 89.2% of the overall microbial population in seeds.

**Fig. 2:**
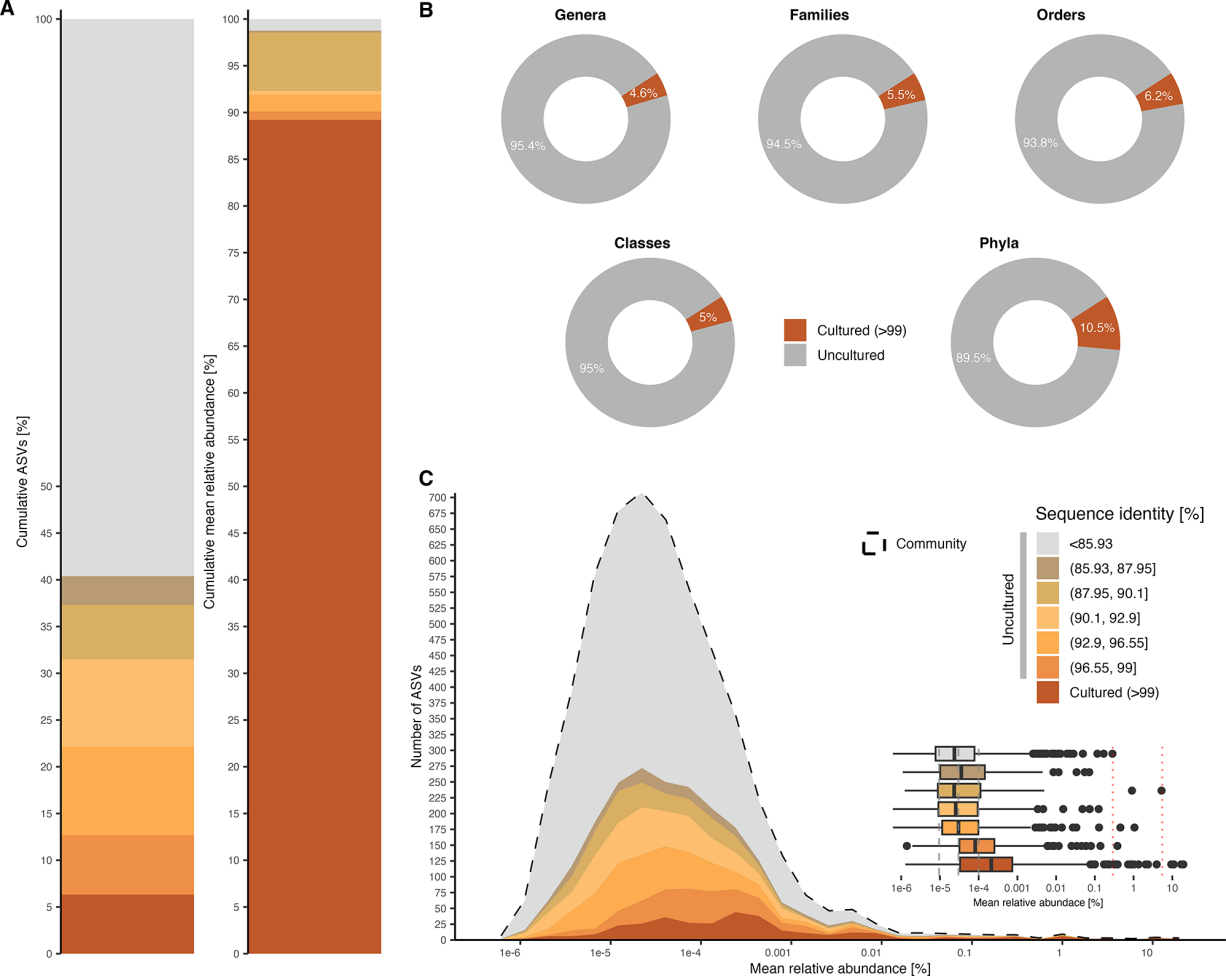
Recovered bacterial endophytic diversity and abundance in the *Cannabis* seed microbiome. **A** Cumulative percentage of community amplicon sequence variants (ASVs) and their mean relative abundance according to sequence identity to the cultured isolates. **B** Proportion of taxonomic ranks (genera, families, orders, classes, and phyla) in the community dataset that are represented in the cultured fraction (> 99% sequence identity). **C** Cumulative community ASV counts based on sequence identity to the isolates and according to mean relative abundance. The inset boxplot shows the distribution of the mean relative abundance of ASVs according to the proposed sequence identity thresholds, highlighting the representation of highly abundant taxa in the cultured fraction

A multiple alignment of ASVs from the community and cultured datasets identified 2513 matching community ASVs out of 5297, with percentage identities ranging from 100 to 77.35% (Fig. S4). Among these, 374 ASVs with sequence identity below 85.93% were classified as phylogenetically distant, and along with non-matching ASVs (*n* = 2784), amounted to 59.62% of the community ASVs (Fig. 2A). The cultured fraction was identified at species level using a sequence identity threshold of > 99%, revealing that only 6.32% of the community ASVs were at least 99% similar to a cultured representative. Despite the low ASV representation, the cultured fraction represented 89.2% of the relative abundance of the community dataset. Collectively, phylogenetically close uncultured community ASVs (> 85.93% sequence identity) made up to 9.56% of the relative abundance.

The cultured fraction represented 4.6% of the genera, 5.5% of the families, 6.2% of the orders, 5% of the classes, and 10.5% of the phyla present in the community dataset (Fig. 2B). The mean relative abundance distribution of the cultured and uncultured fractions showed significant differences (*P* = 2.2e-16, Mann-Whitney U) (Fig. 2C; Table S4). The inset figure showed that taxa without cultured representatives at species level only occurred at mean relative abundances lower than 5.28%, and phylogenetically distant ASVs occurred at mean relative abundances below 0.28%.

### Isolates cover only the five most abundant bacterial classes

**Fig. 3:**
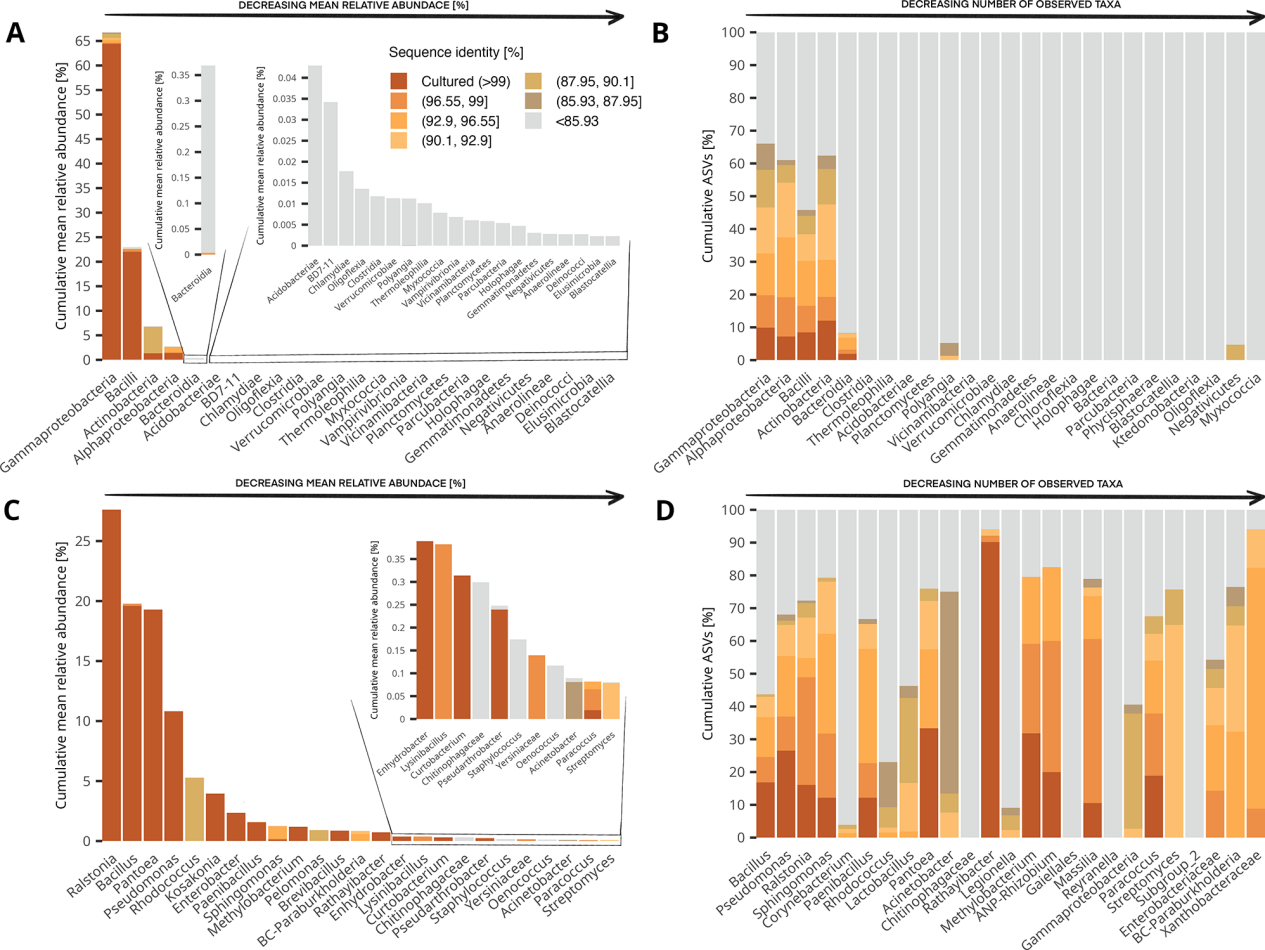
The top 25 *Cannabis* seed bacterial classes and genera, regarding their abundance and diversity in the community dataset, and their missing fractions. **A** Mean relative abundance of the different bacterial classes ranked from higher to lower and showing the cumulative contribution of the cultured and uncultured fractions for each. **B** Diversity of the different bacterial classes ranked from higher to lower and showing the cumulative contribution of the cultured and uncultured fractions for each. **C** Mean relative abundance of the different bacterial genera ranked from higher to lower and showing the cumulative contribution of the cultured and uncultured fractions for each. **D** Diversity of the different bacterial genera ranked from higher to lower and showing the cumulative contribution of the cultured and uncultured fractions for each.

We investigated missing fractions from the *Cannabis* seed microbiome from the 25 most abundant bacterial classes in terms of relative abundance from the community dataset (Fig. 3A). Within the most abundant classes — *Gammaproteobacteria*, *Bacilli*, *Actinobacteria*, and *Alphaproteobacteria* — uncultured fractions of phylogenetically distant ASVs (< 85.93 sequence identity) were residual; but while the cultured fractions of *Gammaproteobacteria* and *Bacilli* were well above 95% of their relative abundance, only 50% of the *Alphaproteobacteria*’s relative abundance shared more than 99% identity with the recovered isolates (Fig. S5A). Moreover, the vast majority of *Actinobacteria*’s relative abundance (76.9%) only matched the cultured taxa at sequence similarities between 90.1% and 87.95%. None of the other classes from the 25 most abundant had cultured fractions nor any other fractions with close similarities to the isolates, except for *Bacteroidia* (0.5%). The 25 most diverse classes from the community dataset ranged from 1367 ASVs in *Gammaproteobacteria* to 20 ASVs in *Myxococcia*. *Gammaproteobacteria*, *Bacilli*, *Actinobacteria* and *Alphaproteobacteria* and *Bacteroidia*, were the only ones that harbored a cultured fraction of ASVs (Fig. 3B). The remaining classes from this group were dominated by uncultured fractions of phylogenetically distant ASVs; exceptions included relatively few ASVs from *Polyangia* and *Negativicutes* (Fig. S5B).

The 25 most abundant and diverse genera belong, in their majority, to the five most abundant classes, except for unclassified members of *Gaiellales* (Thermoleophilia) and Subgroup_2 (*Acidobacteriae*). More than half of the genera within the 25 most abundant ones (*n* = 13) have cultured fractions representing more than 95% of their relative abundance (Fig. 3C); only *Staphylococcus*, *Oenococcus* and members of *Chitiniphagaceae* lacked cultured fractions or uncultured fractions of ASVs phylogenetically closer to the isolates (Fig. S6A). The 25 most diverse genera ranged from 258 ASVs in *Bacillus* to 34 ASVs in members of *Xanthobacteriaceae*. Among them, *Corynebacterium*, *Reynarella*, and members of *Chitiniphagacea*, *Gaiellales*, and Subgroup_2 had more than 95% of uncultured fractions of ASVs phylogenetically distant from the isolates (Fig. S6B). Despite being one of the most cultured genera, 56.2% of *Bacillus* ASVs also belonged to the phylogenetically distant uncultured fraction. Contrasting, ASVs matching *Rathayibacter* isolates with 99% sequence identity cover 90.2% of the total of the genus in the community dataset (Fig. 3D).

### Cultured taxa are peripheral and have lower connectivity

**Fig. 4:**
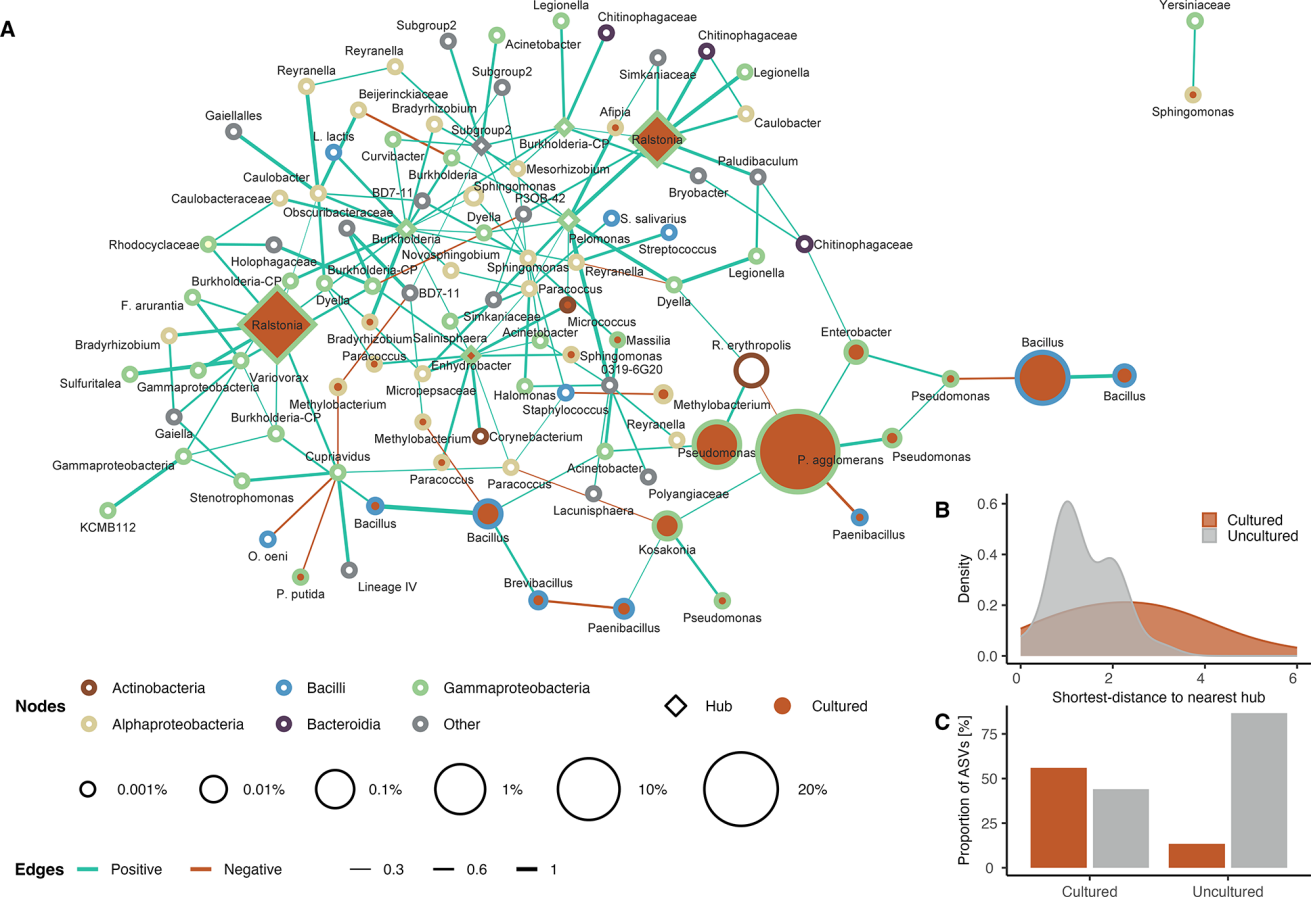
Co-occurrence network and community structure of the *Cannabis* seed microbiota. **A** The network illustrates significant associations (α = 0.05) between amplicon sequence variants (ASVs) as nodes, with taxonomic annotations provided for each ASV. Nodes are colored by bacterial class and their size corresponds to their mean relative abundance. Diamond-shaped nodes represent hubs and orange-filled nodes indicate cultured ASVs (> 99% sequence identity). Edge colors signify association types: green for positive (co-occurrence) and red for negative (mutual exclusion). Edge widths are proportional to association weights, highlighting the strength of connections. **B** Density plot showing the distribution of ASVs based on their shortest distance to the nearest hub, comparing cultured (orange) and uncultured (grey) fractions. **C** Bar plot depicting the proportions of ASVs from cultured and uncultured taxa that share more than 50% of their edges with either cultured or uncultured taxa, emphasizing the degree of association overlap between these groups

We used co-occurrence networks to explore putative interactions in *Cannabis* seeds. The generated network features 103 ASVs from the community dataset with significant associations (*n* = 151), which were mainly positive (90.73%, *n* = 137) (Fig. 4A). The majority of nodes (*n* = 85) were represented by members of the 5 most predominant classes in the community dataset (*Actinobacteria*, *Alphaproteobacteria*, *Bacilli*, *Bacteroidia* and *Gammaproteobacteria*), while a smaller fraction of ASVs (*n* = 18) represented 11 other bacterial classes. Members of the cultured fraction exclusively belonged to *Actinobacteria* (*n* = 1), *Alphaproteobacteria* (*n* = 9), *Bacilli* (*n* = 7) and *Gammaproteobacteria* (*n* = 12). A total of 7 hubs were identified, from which only 3 were cultured (> 99% sequence identity), belonging to the genera *Ralstonia* (*n* = 2) and *Enhydrobacter* (*n* = 1). Uncultured hubs were represented by the genera *Burkholderia-Caballeronia-Paraburkholderia* (*n* = 2), *Pelomonas* (*n* = 1), and an ASV member of Subgroup_2 (*Acidobacteriae*).

We compared different centralities between cultured (28.16%, *n* = 28) and uncultured (71.84%, *n* = 73) taxa connected in the network. We detected a significantly lower closeness centrality (*P* = 0.007, Mann-Whitney U), neighborhood connectivity (*P* = 0.0038, Mann-Whitney U) and radiality (*P* = 0.007, Mann-Whitney U) in the cultured taxa, as well as a significantly higher average shortest path length (*P* = 0.007, Mann-Whitney U), (Fig. S8). We calculated the shortest distance to the nearest hub for both fractions, revealing a significantly higher proximity between hubs and uncultured taxa (*P* = 0.00075, Mann-Whitney U) (Fig. 4B). Lastly, we observed a higher proportion of uncultured nodes co-occurring with other uncultured nodes, than with cultured nodes (> 50% shared edges; *P* ≤ 0.05, χ2) (Fig. 4C). Principal component analysis (PCA) revealed that measures of ecological connectivity and network positioning exert a greater influence than relative abundance in distinguishing cultured from uncultured ASVs within the co-occurrence network (Figure S7).

## Discussion

Research on the seed microbiome has gained momentum in recent years, partly because its role in seed viability and early plant establishment [67], which could be leveraged to improve plant growth and resilience [12]. Currently, there is a renewed interest in culture-based research approaches due to their complementarity to widespread -omics approaches [68]. Here, we put the *Cannabis* seed microbiome into perspective, by holistically exploring the extension of its cultured and uncultured bacterial fractions as well as the factors influencing endophyte culturability.

With our approach, we successfully isolated 36 distinct bacterial genera from *Cannabis* seeds. Previous efforts, focused on seed embryos from three *Cannabis* genotypes (Anka, CRS-1 and Yvonne — not included in this study or in [28]), recovered 19 bacterial strains using NA and LB media [30]. These isolates were primarily assigned to the genera *Pantoea* (37%), *Staphylococcus* (21%), *Bacillus* (16%), *Enterobacter* (16%), *Brevibacterium* (5%), and *Pseudomonas* (5%). While our approach yielded a broader diversity of cultured genera, *Staphylococcus* was notably absent, possibly due to genotypic differences between the accessions used in the two studies. In the community dataset, *Staphylococcus* was detected only sporadically and at low abundance, which suggests ecological exclusion or niche competition [28], ultimately limiting its cultivation. Our culturing efforts yielded exclusively bacteria from the most predominant and highly abundant bacterial classes in the *Cannabis* seed microbiome, (i.e., *Gammaproteobacteria*, *Bacilli*, *Actinobacteria*, *Alphaproteobacteria*, and *Bacteroidia*). The implemented approach encompassed several of the recommendations for obtaining recalcitrant endophytic microorganisms, including soaking and germination of the seeds to activate dormant microorganisms, and the use of serial dilutions and a wide range of nutrient media with prolonged incubation times. These included media supplemented with *Cannabis* extracts aimed at facilitating the isolation of slow-growing bacteria, and to cover nutritional requirements of potentially highly adapted bacteria [69]. While our culture-based dataset is dominated by highly abundant genera, we emphasize that developing low-nutrient or customized media tailored to specific microbial nutrient preferences represents a critical direction for future research. Such methodological refinements could be guided by nutrient profiling approaches with seeds to design media that mimic their specific chemical environment, or to test possible growth inhibiting compounds that might interfere with culturing of seed endophytes.

In our study, over 90% of the isolates matched ASVs detected in the community dataset with > 99% sequence identity. This high concordance is particularly notable given that culturing was performed with different individual seeds, suggesting that certain microbial taxa are consistently detectable, despite individual variation. However, it is important to note that functional differences can exist even at species level [70], meaning that phylogenetic similarity, particularly when inferred from a single variable region of the 16S rRNA gene, may not necessarily translate into functional similarity. A genome level analysis would provide further insights into the functional gene content of microbial communities and elucidate ecological roles of both cultured and uncultured taxa. The cultured fraction represented only a small portion of the total diversity, accounting for 6.32% of all detected ASVs, which represents an even lower recovery rate than typically reported in other oligotrophic environments [6]. However, comparisons across studies must be interpreted with caution, as differences in the approaches used to classify cultured versus uncultured taxa can significantly influence diversity estimates. To go beyond a rigid binary classification, we defined different degrees for the ‘uncultured status’ that is based on sequence identity. We found that 59.62% of the missing diversity consists of phylogenetically distant (< 85.93% sequence identity), uncultured bacteria. These members of the *Cannabis* seed microbiome likely possess unique physiologies that may require novel culturing strategies to recover. Uncovering these taxa could be highly valuable, as they may provide yet undiscovered functions for the *Cannabis* holobiont.

We revealed that bacterial abundance has a major impact on culturing but is independent of the diversity within each genus or class. Rare taxa are often associated with so-called ‘microbiome dark matter’, and the contribution of these microorganisms has been proposed to be conducive to the stability and function of the microbiome [71, 72]. In our analysis, the uncultured fraction of the rare microbiota conveys important classes such as *Acidobacteriae* or *Verrucomicrobiae*, which are known rhizosphere oligotrophs with members associated with plant growth promoting capabilities [73–75]. The most abundant, non-recovered ASVs belonged to *Actinobacteria*, *Alphaproteobacteria*, and *Bacteroidia*. These bacterial classes are very diverse, and it is possible that some of their uncultured seed microbiome members hold important functions. Further, abundant taxa classified as *Rhodococcus* and *Pelomonas* did not have cultured representatives but had high similarities to cultured taxa. Further investigation of them could prove worthy as some representatives have been utilized for bioremediation and nitrogen fixation, respectively [76, 77]. This contrasts with rare genera like *Staphylococcus*,* Oenococcus*, and members of *Chitinophagaceae*, whose members have no matching ASVs to the cultured taxa. While there is a clear tendency favoring more abundant taxa, we still retrieved some representatives of low abundant genera like *Paracoccus* or *Massilia*, known for its antagonistic properties [78]. Lastly, despite the available knowledge about significant functional and ecological roles of seed core bacterial taxa such as *Pantoea*, *Pseudomonas*, and *Enterobacter*, three out of the seven representative genera from the *Cannabis* seed core microbiome remained underrepresented, including *Pelomonas*, *Rhodococcus*, and *Burkholderia* [28].

Co-occurrence network analysis of the *Cannabis* seed microbiome revealed clear distinctions between cultured and uncultured taxa, suggesting that ecological interactions, rather than dominance alone, are critical factors shaping the likelihood of successful microbial isolation. We observed a higher proportion of co-occurrence among uncultured taxa, with cultured taxa occupying predominantly peripheral positions within the network. This feature has been previously associated with abundant microbes [79], however, this relationship appears to be environment-dependent [80], and our data did not support a direct link between abundance and network positioning. This strong association between high network centrality and uncultured taxa likely reflects metabolic dependencies on other seed-borne microbes [81]. Microbial interactions can take many forms: from physical contact to syntrophic relationships, involving dependencies on amino acids, vitamins or other critical metabolites that ultimately contribute to microbial recalcitrance to culturing [82, 83]. Further supporting this, we observed higher radiality and neighborhood connectivity in the uncultured fraction, indicative of greater network accessibility and more efficient transfer between nodes [84]. These ecological patterns lay a foundation for future co-culture or synthetic community experiments designed to overcome cultivation barriers and dissect specific interactions under controlled conditions to identify causal relationships.

We highlighted putative dependencies from hubs in uncultured taxa, which are missing in taxa with cultured representatives. Hub microorganisms can be key in recovering the uncultured fraction due to their roles in connecting different parts of the network [79]. In other environments, approaches using microbial hubs to facilitate the culturing of microorganisms have shown positive outcomes [80, 84]. However, we also report highly abundant cultured hubs that may act as deterrents to the culturing of taxa that frequently co-occur with them. This observation supports previous hypothesis proposing that dominance and competition are major forces shaping the culturability of seed-associated microorganisms [41]. In this context, the positive associations between those nodes likely reflect shared environmental preferences rather than true metabolic dependencies. It is possible that certain uncultured microorganisms persist in the community through niche partitioning or community buffering effects. Such taxa may require more targeted strategies, such as dilution-to-extinction approaches, to overcome competitive exclusion and achieve successful isolation [85]. Further, the presence of potentially phytopathogenic bacteria such as *Ralstonia* and *Klebsiella* in healthy *Cannabis* seedlings suggests that they may adopt different behaviors under specific ecological contexts, depending on host status, microbiome composition, or environmental cues [86]. It is possible that their activity is suppressed by other members of the microbiome through competitive exclusion, antibiosis, or quorum quenching, as previously demonstrated in seed microbiome studies such as that of Matsumoto *et al.* [87]. This coexistence highlights the need for context-specific evaluations of microbial functions, rather than assumptions based solely on taxonomy. Understanding how these potentially pathogenic genera coexist with beneficial microbes in the seed could offer valuable insights into natural disease suppression mechanisms and the functional plasticity of seed endophytes.

The overall results highlight the importance of integrating ecological insights into strategies for seed microbiome culturing. Culturing of seed-associated microorganisms will allow for a deeper understanding of the functional roles played by seed endophytes in *Cannabis* health and open new pathways for optimizing microbiome-based breeding approaches, aligning with the goals of sustainable and precision agriculture. This potential is exemplified in our previous study based on *Cannabis* plants [28] in which reintroduction of a seed endophyte lost through domestication significantly improved plant growth, demonstrating the practical benefits of harnessing native microbial allies in breeding and management programs.

## Conclusion

Seed-associated microorganisms represent a valuable reservoir of beneficial microbes and serve as strategic targets for microbiome-based breeding approaches. Their inherent adaptation to the host plant makes them well-suited for *in planta* establishment, offering the potential to enhance plant health and performance without disrupting native microbial communities. Our study provides new insights into the culturable and uncultured fractions of the *Cannabis* seed microbiome, highlighting key microbial groups that remain recalcitrant to cultivation and the factors influencing *Cannabis* seed endophytic culturability. While we successfully recovered members of the most abundant bacterial classes, a substantial portion of the microbiome, including rare and potentially functionally significant taxa, remained uncultured. This highlights methodological refinements in culturing approaches as a critical direction for advancing seed microbiome research. Network analysis revealed that uncultured taxa exhibited higher connectivity, and stronger associations with microbial hub nodes, suggesting ecological dependencies influencing their culturability. These findings underscore the putative role of microbial interactions in shaping seed communities and offer new perspectives on leveraging microbial networks to enhance cultivation strategies in seed microbiomes. The identification of hub taxa as potential facilitators for recovering uncultured microorganisms aligns with previous approaches that have successfully used network-informed culturing techniques, but competition may also favor dominant microorganisms. While culturing can aid in understanding the ecological roles of these microbes, particularly in relation to plant health and resilience, this framework could provide new opportunities for microbiome-assisted improvements in *Cannabis* cultivation.

## Electronic supplementary material

Below is the link to the electronic supplementary material.

Supplementary Material 1

Supplementary Material 2

## Data Availability

The 16S rRNA V4 gene region dataset obtained from *Cannabis* seed endophytic bacterial communities was retrieved from the European Nucleotide Archive (ENA; https://www.ebi.ac.uk/ena) under the accession number PRJEB64469. The 16S rRNA V1-V9 gene region dataset obtained from *Cannabis* seed endophytic isolates was deposited in ENA under the accession number PRJEB83654. The analysis pipeline and code for the figures are available in the GitHub repository, https://github.com/cbclobato/culturing-seed-bacteria.

## References

[1] Pace NR. Mapping the Tree of Life: Progress and Prospects, Microbiol Mol Biol Rev. Dec. 2009;73(4):565–576. 10.1128/MMBR.00033-09 10.1128/MMBR.00033-09 PMC2786576 19946133

[2] Rinke C et al. Jul., Insights into the phylogeny and coding potential of microbial dark matter, Nature. 2013;499(7459):431–437. 10.1038/nature12352 10.1038/nature12352 23851394

[3] Sarhan MS, et al. Culturomics of the plant prokaryotic Microbiome and the dawn of plant-based culture media– A review. J Adv Res. Sep. 2019;19:15–27. 10.1016/j.jare.2019.04.002. 10.1016/j.jare.2019.04.002 PMC6630032 31341666

[4] Giovannoni S, Stingl U. The importance of culturing bacterioplankton in the ‘omics’ age, Nat Rev Microbiol. Oct. 2007;5(10):820–826. 10.1038/nrmicro1752 10.1038/nrmicro1752 17853909

[5] Hug LA, et al. A new view of the tree of life. Nat Microbiol. Apr. 2016;1(5):16048. 10.1038/nmicrobiol.2016.48. 10.1038/nmicrobiol.2016.48 27572647

[6] Lloyd KG, Steen AD, Ladau J, Yin J, Crosby L. Oct., Phylogenetically Novel Uncultured Microbial Cells Dominate Earth Microbiomes. mSystems. 2018;3(5). 10.1128/msystems.00055-18 10.1128/mSystems.00055-18 PMC6156271 30273414

[7] Martiny AC. High proportions of bacteria are culturable across major biomes. ISME J. Aug. 2019;13(8):2125–8. 10.1038/s41396-019-0410-3. 10.1038/s41396-019-0410-3 PMC6775996 30952994

[8] Youseif SH et al. Oct., Comparative Analysis of the Cultured and Total Bacterial Community in the Wheat Rhizosphere Microbiome Using Culture-Dependent and Culture-Independent Approaches. Microbiol Spectr. 2021;9(2):e00678-21. 10.1128/Spectrum.00678-21 10.1128/Spectrum.00678-21 PMC8528112 34668733

[9] Müller T, Ruppel S. Progress in cultivation-independent phyllosphere microbiology. FEMS Microbiol Ecol. Jan. 2014;87(1):2–17. 10.1111/1574-6941.12198. 10.1111/1574-6941.12198 PMC3906827 24003903

[10] Thomas P, Shaik SP. Molecular profiling on Surface-Disinfected tomato seeds reveals high diversity of Cultivation-Recalcitrant endophytic Bacteria with low shares of Spore-Forming Firmicutes. Microb Ecol. May 2020;79(4):910–24. 10.1007/s00248-019-01440-5. 10.1007/s00248-019-01440-5 31720799

[11] Shahzad R, Khan AL, Bilal S, Asaf S, Lee I-J. What is there in seeds?? Vertically transmitted endophytic resources for sustainable improvement in plant growth. Front Plant Sci. Jan. 2018;9:24. 10.3389/fpls.2018.00024. 10.3389/fpls.2018.00024 PMC5787091 29410675

[12] Abdelfattah A, Tack AJM, Lobato C, Wassermann B, Berg G. From seed to seed: the role of microbial inheritance in the assembly of the plant Microbiome. Trends Microbiol. Apr. 2023;31(4):346–55. 10.1016/j.tim.2022.10.009. 10.1016/j.tim.2022.10.009 36481186

[13] Simonin M, et al. Seed microbiota revealed by a large-scale meta-analysis including 50 plant species. Microbiol Preprint Jun. 2021. 10.1101/2021.06.08.447541. 10.1111/nph.18037 35175621

[14] Shade A, Jacques M-A, Barret M. Ecological patterns of seed Microbiome diversity, transmission, and assembly. Curr Opin Microbiol. Jun. 2017;37:15–22. 10.1016/j.mib.2017.03.010. 10.1016/j.mib.2017.03.010 28437661

[15] Bergmann GE, Leveau JHJ. A metacommunity ecology approach to Understanding microbial community assembly in developing plant seeds. Front Microbiol. Jul. 2022;13:877519. 10.3389/fmicb.2022.877519. 10.3389/fmicb.2022.877519 PMC9355165 35935241

[16] Hardoim P. The Ecology of Seed Microbiota, in Seed Endophytes, S. K. Verma and J. F. White, Jr, Eds., Cham: Springer International Publishing, 2019, pp. 103–125. 10.1007/978-3-030-10504-4_6

[17] Bziuk N, et al. The treasure inside barley seeds: microbial diversity and plant beneficial bacteria. Environ Microbiome. Dec. 2021;16(1). 10.1186/s40793-021-00389-8. 10.1186/s40793-021-00389-8 PMC8554914 34711269

[18] Hone H, et al. Profiling, isolation and characterisation of beneficial microbes from the seed microbiomes of drought tolerant wheat. Sci Rep. Jun. 2021;11(1):11916. 10.1038/s41598-021-91351-8. 10.1038/s41598-021-91351-8 PMC8184954 34099781

[19] López JL, et al. Isolation, taxonomic analysis, and phenotypic characterization of bacterial endophytes present in alfalfa (Medicago sativa) seeds. J Biotechnol. Feb. 2018;267:55–62. 10.1016/j.jbiotec.2017.12.020. 10.1016/j.jbiotec.2017.12.020 29292130

[20] Links MG, Demeke T, Gräfenhan T, Hill JE, Hemmingsen SM, Dumonceaux TJ. Simultaneous profiling of seed-associated bacteria and fungi reveals antagonistic interactions between microorganisms within a shared epiphytic microbiome on T riticum and B rassica seeds. New Phytol. Apr. 2014;202(2):542–553. 10.1111/nph.12693 10.1111/nph.12693 PMC4235306 24444052

[21] Bertani I, Abbruscato P, Piffanelli P, Subramoni S, Venturi V. Rice bacterial endophytes: isolation of a collection, identification of beneficial strains and microbiome analysis: Beneficial bacterial endophytes of rice. Environmental Microbiology Reports. Jun. 2016;8(3):388–398. 10.1111/1758-2229.12403 10.1111/1758-2229.12403 27038229

[22] Wolfgang A, et al. Understanding the impact of cultivar, seed origin, and substrate on bacterial diversity of the sugar beet rhizosphere and suppression of Soil-Borne pathogens. Front Plant Sci. Sep. 2020;11:560869. 10.3389/fpls.2020.560869. 10.3389/fpls.2020.560869 PMC7554574 33101330

[23] Rybakova D, et al. The structure of the Brassica napus seed Microbiome is cultivar-dependent and affects the interactions of symbionts and pathogens. Microbiome. Dec. 2017;5(1):104. 10.1186/s40168-017-0310-6. 10.1186/s40168-017-0310-6 PMC5580328 28859671

[24] Adam E, Bernhart M, Müller H, Winkler J, Berg G. The Cucurbita pepo seed microbiome: genotype-specific composition and implications for breeding. Plant Soil. Jan. 2018;422:1–2. 10.1007/s11104-016-3113-9.

[25] Michl K, et al. Determining the footprint of breeding in the seed Microbiome of a perennial cereal. Environ Microbiome. Jun. 2024;19(1):40. 10.1186/s40793-024-00584-3. 10.1186/s40793-024-00584-3 PMC11184768 38886863

[26] Wentzien NM, et al. Pitting the Olive seed Microbiome. Environ Microbiome. Mar. 2024;19(1). 10.1186/s40793-024-00560-x. 10.1186/s40793-024-00560-x PMC10943921 38491515

[27] Compant S, Mitter B, Colli-Mull JG, Gangl H, Sessitsch A. Endophytes of Grapevine Flowers, Berries, and Seeds: Identification of Cultivable Bacteria, Comparison with Other Plant Parts, and Visualization of Niches of Colonization. Microb Ecol. Jul. 2011;62(1):188–197. 10.1007/s00248-011-9883-y 10.1007/s00248-011-9883-y 21625971

[28] Lobato C, De Freitas JM, Habich D, Kögl I, Berg G, Cernava T. Wild again: recovery of a beneficial Cannabis seed endophyte from low domestication genotypes. Microbiome. Nov. 2024;12(1):239. 10.1186/s40168-024-01951-5. 10.1186/s40168-024-01951-5 PMC11568533 39548475

[29] Davies J, Hawkins S, Winters A, Farrar K. Bacterial endophytic community composition varies by hemp cultivar in commercially sourced seed. Environ Microbiol Rep. Apr. 2024;16(2):e13259. 10.1111/1758-2229.13259. 10.1111/1758-2229.13259 PMC11035101 38649235

[30] Scott M, Rani M, Samsatly J, Charron J-B, Jabaji S. Endophytes of industrial hemp Cannabis sativa L.) cultivars: identification of culturable bacteria and fungi in leaves, petioles, and seeds. Can. J. Microbiol. Oct. 2018;64(10):664–680. 10.1139/cjm-2018-0108 10.1139/cjm-2018-0108 29911410

[31] Thomas P, Sahu PK. Vertical transmission of diverse Cultivation-Recalcitrant endophytic Bacteria elucidated using watermelon seed embryos. Front Microbiol. Nov. 2021;12:635810. 10.3389/fmicb.2021.635810. 10.3389/fmicb.2021.635810 PMC8634838 34867834

[32] Gerna D, Clara D, Allwardt D, Mitter B, Roach T. Tailored Media Are Key to Unlocking the Diversity of Endophytic Bacteria in Distinct Compartments of Germinating Seeds. Microbiol Spectr. Aug. 2022;10(4):e00172-22. 10.1128/spectrum.00172-22 10.1128/spectrum.00172-22 PMC9431621 35867396

[33] Bergmann GE, Heitmann SJ, Busby PE, Leveau JHJ. Characterization of Seed Mycobiota Using Culture-Dependent and Culture-Independent Approaches, in Microbial Environmental Genomics (MEG), vol. 2605, F. Martin and S. Uroz, Eds., in Methods in Molecular Biology, vol. 2605., New York, NY: Springer US, 2023, pp. 65–78. 10.1007/978-1-0716-2871-3_4 10.1007/978-1-0716-2871-3_4 36520389

[34] Chesneau G, et al. Temporal dynamics of bacterial communities during seed development and maturation. FEMS Microbiol Ecol. Nov. 2020;96(12):fiaa190. 10.1093/femsec/fiaa190. 10.1093/femsec/fiaa190 PMC8096252 32966572

[35] Truyens S, Weyens N, Cuypers A, Vangronsveld J. Bacterial seed endophytes: genera, vertical transmission and interaction with plants: Bacterial seed endophytes. Environmental Microbiology Reports. Feb. 2015;7(1):40–50. 10.1111/1758-2229.12181

[36] Barer MR, Harwood CR. Bacterial viability and culturability. in Advances in microbial physiology. Volume 41. Elsevier; 1999. pp. 93–137. 10.1016/S0065-2911(08)60166-6. 10.1016/s0065-2911(08)60166-6 10500845

[37] Dong K et al. Jan., Induction, detection, formation, and resuscitation of viable but non-culturable state microorganisms. Comp Rev Food Sci Food Safe. 2020;19(1):149–183. 10.1111/1541-4337.12513 10.1111/1541-4337.12513 33319518

[38] Okunishi S, Sako K, Mano H, Imamura A, Morisaki H. Bacterial flora of endophytes in the maturing seed of cultivated rice (Oryza sativa). Microb Environ. 2005;20(3):168–77. 10.1264/jsme2.20.168.

[39] Cope-Selby N, Cookson A, Squance M, Donnison I, Flavell R, Farrar K. Endophytic bacteria in Miscanthus seed: implications for germination, vertical inheritance of endophytes, plant evolution and breeding. GCB Bioenergy. Jan. 2017;9(1):57–77. 10.1111/gcbb.12364.

[40] Mano H, Tanaka F, Watanabe A, Kaga H, Okunishi S, Morisaki H. Culturable surface and endophytic bacterial flora of the maturing seeds of rice plants (Oryza sativa) cultivated in a paddy field. Microb Environ. 2006;21(2):86–100. 10.1264/jsme2.21.86.

[41] Newcombe G, Harding A, Ridout M, Busby PE. A hypothetical bottleneck in the plant Microbiome. Front Microbiol. Jul. 2018;9:1645. 10.3389/fmicb.2018.01645. 10.3389/fmicb.2018.01645 PMC6080073 30108556

[42] Yan C, Owen JS, Seo E-Y, Jung D, He S. Microbial Interaction is Among the Key Factors for Isolation of Previous Uncultured Microbes. J Microbiol. Jul. 2023;61(7):655–662. 10.1007/s12275-023-00063-3 10.1007/s12275-023-00063-3 PMC10477116 37589838

[43] Shade A et al. Aug., Conditionally Rare Taxa Disproportionately Contribute to Temporal Changes in Microbial Diversity. mBio. 2014;5(4):e01371-14. 10.1128/mBio.01371-14 10.1128/mBio.01371-14 PMC4161262 25028427

[44] Lima. Demultiplex barcoded samples. PacBio & bioconda. [Online]. Available: lima.how.

[45] Core Team R. R: A language and environment for statistical computing. (2021). R Foundation for Statistical Computing, Vienna, Austria. [Online]. Available: https://www.R-project.org/

[46] Callahan BJ, et al. High-throughput amplicon sequencing of the full-length 16S rRNA gene with single-nucleotide resolution. Nucleic Acids Res. Oct. 2019;47(18):e103–103. 10.1093/nar/gkz569. 10.1093/nar/gkz569 PMC6765137 31269198

[47] Quast C, et al. The SILVA ribosomal RNA gene database project: improved data processing and web-based tools. Nucleic Acids Res. Nov. 2012;41:D590–6. 10.1093/nar/gks1219. no. D1. 10.1093/nar/gks1219 PMC3531112 23193283

[48] Wang Q, Garrity GM, Tiedje JM, Cole JR. Naïve Bayesian Classifier for Rapid Assignment of rRNA Sequences into the New Bacterial Taxonomy. Appl Environ Microbiol. Aug. 2007;73(16):5261–5267. 10.1128/AEM.00062-07 10.1128/AEM.00062-07 PMC1950982 17586664

[49] Callahan BJ, McMurdie PJ, Rosen MJ, Han AW, Johnson AJA, Holmes SP. DADA2: High-resolution sample inference from Illumina amplicon data. Nat Methods. Jul. 2016;13(7):581–583. 10.1038/nmeth.3869 10.1038/nmeth.3869 PMC4927377 27214047

[50] Bolyen E, et al. Reproducible, interactive, scalable and extensible Microbiome data science using QIIME 2. Nat Biotechnol. Aug. 2019;37(8):852–7. 10.1038/s41587-019-0209-9. 10.1038/s41587-019-0209-9 PMC7015180 31341288

[51] Rognes T, Flouri T, Nichols B, Quince C, Mahé F. VSEARCH: a versatile open source tool for metagenomics. PeerJ. Oct. 2016;4:e2584. 10.7717/peerj.2584. 10.7717/peerj.2584 PMC5075697 27781170

[52] Davis NM, Proctor DM, Holmes SP, Relman DA, Callahan BJ. Simple statistical identification and removal of contaminant sequences in marker-gene and metagenomics data. Microbiome. Dec. 2018;6(1):226. 10.1186/s40168-018-0605-2. 10.1186/s40168-018-0605-2 PMC6298009 30558668

[53] McMurdie PJ, Holmes S. PLoS ONE. Apr. 2013;8(4):e61217. 10.1371/journal.pone.0061217. phyloseq: An R Package for Reproducible Interactive Analysis and Graphics of Microbiome Census Data. 10.1371/journal.pone.0061217 PMC3632530 23630581

[54] Wickham H, et al. Welcome to the tidyverse. JOSS. Nov. 2019;4(43):1686. 10.21105/joss.01686.

[55] Cock PJA, Chilton JM, Grüning B, Johnson JE, Soranzo N. NCBI BLAST + integrated into Galaxy. Gigascience. Dec. 2015;4(1):s13742-015-0080–7. 10.1186/s13742-015-0080-7 10.1186/s13742-015-0080-7 PMC4557756 26336600

[56] Zhang Z, Schwartz S, Wagner L, Miller W. A Greedy Algorithm for Aligning DNA Sequences. Journal of Computational Biology. Feb. 2000;7(1–2):203–214. 10.1089/10665270050081478 10.1089/10665270050081478 10890397

[57] Gwak H-J, Rho M. Front Microbiol. Nov. 2020;11:570825. 10.3389/fmicb.2020.570825. Data-Driven Modeling for Species-Level Taxonomic Assignment From 16S rRNA: Application to Human Microbiomes. 10.3389/fmicb.2020.570825 PMC7688474 33262743

[58] Yarza P et al. Sep., Uniting the classification of cultured and uncultured bacteria and archaea using 16S rRNA gene sequences. Nat Rev Microbiol. 2014;12(9):635–645. 10.1038/nrmicro3330 10.1038/nrmicro3330 25118885

[59] Tamura K, Stecher G, Kumar S. MEGA11: Molecular Evolutionary Genetics Analysis Version 11. Molecular Biology and Evolution. Jun. 2021;38(7):3022–7. 10.1093/molbev/msab120 10.1093/molbev/msab120 PMC8233496 33892491

[60] Edgar RC. MUSCLE: multiple sequence alignment with high accuracy and high throughput. Nucleic Acids Research. Mar. 2004;32(5):1792–1797. 10.1093/nar/gkh340 10.1093/nar/gkh340 PMC390337 15034147

[61] Letunic I, Bork P. Interactive tree of life (iTOL) v6: recent updates to the phylogenetic tree display and annotation tool. Nucleic Acids Res. Jul. 2024;52:W78–82. 10.1093/nar/gkae268. no. W1. 10.1093/nar/gkae268 PMC11223838 38613393

[62] Tackmann J, Matias Rodrigues JF, Mering CV. Rapid Inference of Direct Interactions in Large-Scale Ecological Networks from Heterogeneous Microbial Sequencing Data. Cell Systems. Sep. 2019;9(3):286–296.e8. 10.1016/j.cels.2019.08.002 10.1016/j.cels.2019.08.002 31542415

[63] Bezanson J, Edelman A, Karpinski S, Shah VB. Julia: A Fresh Approach to Numerical Computing. SIAM Rev. Jan. 2017;59(1):65–98. 10.1137/141000671

[64] Shannon P et al. Nov., Cytoscape: A Software Environment for Integrated Models of Biomolecular Interaction Networks. Genome Res. 2003;13(11):2498–2504. 10.1101/gr.1239303 10.1101/gr.1239303 PMC403769 14597658

[65] Berry D, Widder S. Deciphering microbial interactions and detecting keystone species with co-occurrence networks. Front Microbiol. May 2014;5. 10.3389/fmicb.2014.00219. 10.3389/fmicb.2014.00219 PMC4033041 24904535

[66] Csárdi G, et al. Igraph for R: R interface of the Igraph library for graph theory and network analysis. (Dec. 2024;10. 10.5281/ZENODO.7682609. Zenodo.

[67] Chesneau G et al. Dec., Single Seed Microbiota: Assembly and Transmission from Parent Plant to Seedling. mBio. 2022;13(6):e01648-22. 10.1128/mbio.01648-22 10.1128/mbio.01648-22 PMC9765463 36222511

[68] Liu S, Moon CD, Zheng N, Huws S, Zhao S, Wang J. Microbiome. May 2022;10(1):76. 10.1186/s40168-022-01272-5. Opportunities and challenges of using metagenomic data to bring uncultured microbes into cultivation. 10.1186/s40168-022-01272-5 PMC9097414 35546409

[69] Eevers N et al. Jul., Optimization of isolation and cultivation of bacterial endophytes through addition of plant extract to nutrient media. Microbial Biotechnology. 2015;8(4):707–715. 10.1111/1751-7915.12291 10.1111/1751-7915.12291 PMC4476825 25997013

[70] Sergaki C, Lagunas B, Lidbury I, Gifford ML, Schäfer P. Challenges and approaches in Microbiome research: from fundamental to applied. Front Plant Sci. Aug. 2018;9:1205. 10.3389/fpls.2018.01205. 10.3389/fpls.2018.01205 PMC6107787 30174681

[71] Lynch MDJ, Neufeld JD. Ecology and exploration of the rare biosphere. Nat Rev Microbiol. Apr. 2015;13(4):217–229. 10.1038/nrmicro3400 10.1038/nrmicro3400 25730701

[72] Pascoal F, Magalhães C, Costa R. The link between the ecology of the prokaryotic rare biosphere and its biotechnological potential. Front Microbiol. Feb. 2020;11:231. 10.3389/fmicb.2020.00231. 10.3389/fmicb.2020.00231 PMC7042395 32140148

[73] Kalam S, Basu A, Podile AR. Difficult-to-culture bacteria in the rhizosphere: the underexplored signature microbial groups. Pedosphere. Feb. 2022;32(1):75–89. 10.1016/S1002-0160(21)60062-0.

[74] Kielak AM, Barreto CC, Kowalchuk GA, Van Veen JA, Kuramae EE. The ecology of acidobacteria: moving beyond genes and genomes. Front Microbiol. May 2016;7. 10.3389/fmicb.2016.00744. 10.3389/fmicb.2016.00744 PMC4885859 27303369

[75] Bünger W, Jiang X, Müller J, Hurek T, Reinhold-Hurek B. Novel cultivated endophytic Verrucomicrobia reveal deep-rooting traits of bacteria to associate with plants. Sci Rep. May 2020;10(1):8692. 10.1038/s41598-020-65277-6. 10.1038/s41598-020-65277-6 PMC7251102 32457320

[76] Ceniceros A, Dijkhuizen L, Petrusma M, Medema MH. Genome-based exploration of the specialized metabolic capacities of the genus Rhodococcus. BMC Genomics. Dec. 2017;18(1):593. 10.1186/s12864-017-3966-1. 10.1186/s12864-017-3966-1 PMC5550956 28793878

[77] Xie C-H, Yokota A. Reclassification of Alcaligenes latus strains IAM 12599T and IAM 12664 and Pseudomonas saccharophila as Azohydromonas lata gen. nov., comb. nov., Azohydromonas australica sp. nov. and Pelomonas saccharophila gen. nov., comb. nov., respectively. International Journal of Systematic and Evolutionary Microbiology. Nov. 2005;55(6):2419–2425. 10.1099/ijs.0.63733-0 10.1099/ijs.0.63733-0 16280506

[78] Lin C-C, et al. Integrated omics approach to unveil antifungal bacterial Polyynes as acetyl-CoA acetyltransferase inhibitors. Commun Biol. May 2022;5(1):454. 10.1038/s42003-022-03409-6. 10.1038/s42003-022-03409-6 PMC9098870 35551233

[79] Xian W-D, et al. Network-directed efficient isolation of previously uncultivated Chloroflexi and related bacteria in hot spring microbial Mats. Npj Biofilms Microbiomes. Apr. 2020;6(1):20. 10.1038/s41522-020-0131-4. 10.1038/s41522-020-0131-4 PMC7190741 32350263

[80] Zamkovaya T, Foster JS, De Crécy-Lagard V, Conesa A. A network approach to elucidate and prioritize microbial dark matter in microbial communities. ISME J. Jan. 2021;15(1):228–44. 10.1038/s41396-020-00777-x. 10.1038/s41396-020-00777-x PMC7852563 32963345

[81] Zelezniak A, Andrejev S, Ponomarova O, Mende DR, Bork P, Patil KR. Metabolic dependencies drive species co-occurrence in diverse microbial communities. Proc. Natl. Acad. Sci. U.S.A. May 2015;112(20):6449–6454. 10.1073/pnas.1421834112 10.1073/pnas.1421834112 PMC4443341 25941371

[82] Stewart EJ, Bacteria GU. Bacteriol J. Aug. 2012;194(16):4151–60. 10.1128/JB.00345-12 10.1128/JB.00345-12 PMC3416243 22661685

[83] Rose CJ, Rainey PB. Cooperation and public goods, bacterial. in Encyclopedia of evolutionary biology. Elsevier; 2016. pp. 374–80. 10.1016/B978-0-12-800049-6.00234-1.

[84] Jousset A, et al. Where less May be more: how the rare biosphere pulls ecosystems strings. ISME J. Apr. 2017;11(4):853–62. 10.1038/ismej.2016.174. 10.1038/ismej.2016.174 PMC5364357 28072420

[85] Ordonez A, Hussain U, Cambon MC, Golyshin PN, Downie J, McDonald JE. Evaluating agar-plating and dilution-to-extinction isolation methods for generating oak-associated microbial culture collections. ISME Commun. Jan. 2025;5(1):ycaf019. 10.1093/ismeco/ycaf019. 10.1093/ismeco/ycaf019 PMC11878766 40041709

[86] Hassani MA, Durán P, Hacquard S. Microbial interactions within the plant holobiont. Microbiome. Dec. 2018;6(1):58. 10.1186/s40168-018-0445-0. 10.1186/s40168-018-0445-0 PMC5870681 29587885

[87] Matsumoto H, et al. Bacterial seed endophyte shapes disease resistance in rice. Nat Plants. Jan. 2021;7(1):60–72. 10.1038/s41477-020-00826-5. 10.1038/s41477-020-00826-5 33398157

