# Supplementary material for: Defining the cultured and uncultured bacterial fractions in Cannabis seeds
Source: Environ Microbiome. 2025 Jun 11;20:68. doi: 10.1186/s40793-025-00731-4 (PMC12160407; doi:10.1186/s40793-025-00731-4)
Supplement: Supplementary file 1 — Supplementary Material 1 [file 40793_2025_731_MOESM1_ESM.docx]

# **Supporting Information**

# **Defining the cultured and uncultured bacterial fractions in *Cannabis* seeds**

Carolina Lobato^1^, Ahmed Abdelfattah^2^, Gabriele Berg^1,2,3^, and Tomislav Cernava^1,4^

^1^ Institute of Environmental Biotechnology, Graz University of Technology, Petersgasse 12, 8010 Graz, Austria.

^2^ Leibniz Institute for Agricultural Engineering and Bioeconomy, Max-Eyth-Allee 100, 14 469 Potsdam, Germany.

^3^ Institute for Biochemistry and Biology, University of Potsdam, Karl-Liebknecht-Str. 24-25, 14 476 Potsdam OT Golm, Germany

^4^ School of Biological Sciences, Faculty of Environmental and Life Sciences, Highfield Campus, SO17 1BJ Southampton, UK

**Corresponding author:** tomislav.cernava@tugraz.at | +43 316 873 8312


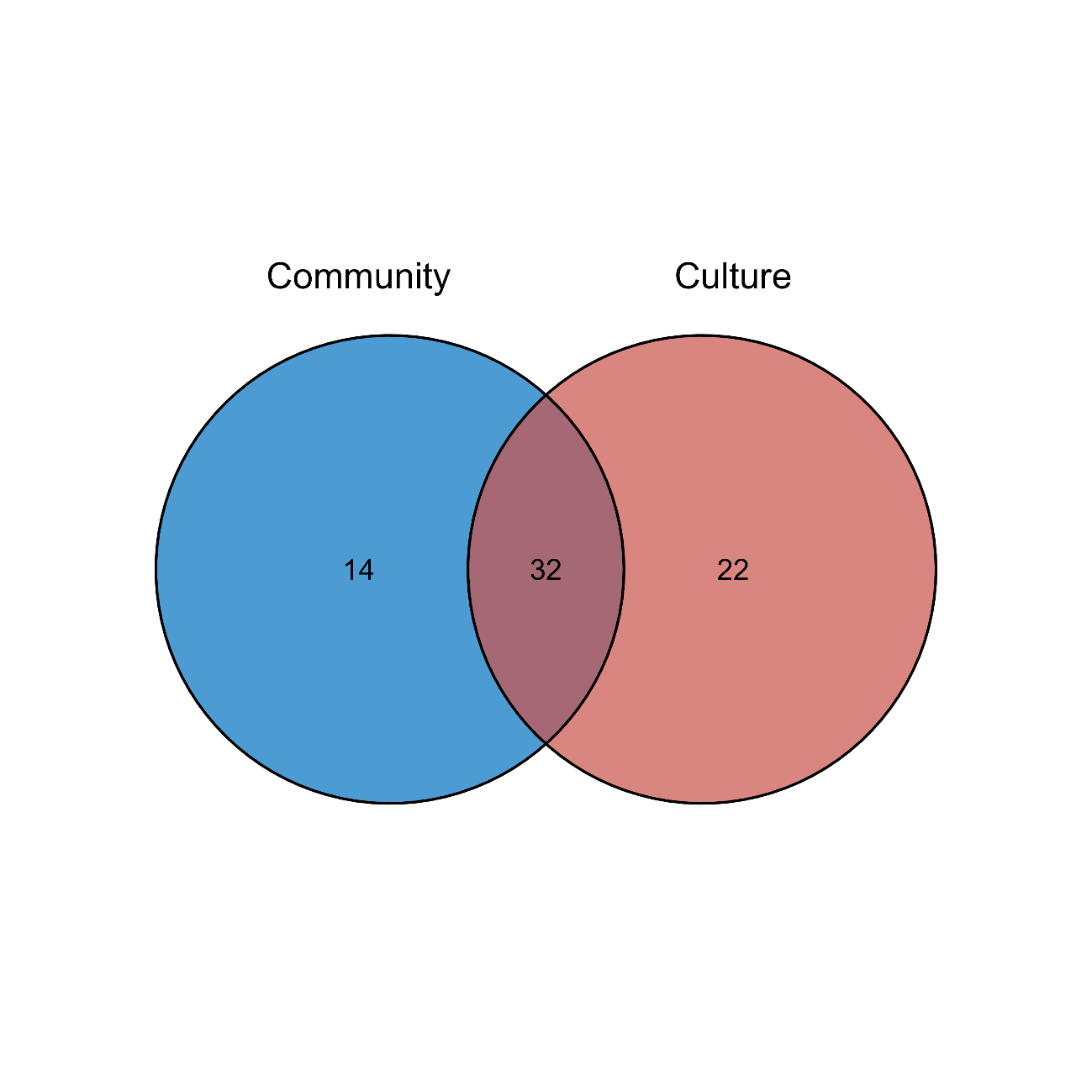


**Figure S1** Unique and overlapping *Cannabis* seed accessions between this study and Lobato et al. 2024. Community refers to amplicon sequencing-based analyses and culture to the cultivation-based approach.





**Figure S2** Mock and PCR negative control samples in the cultured dataset. Two *Sphingomonas* ASVs (ASV 94 and ASV 111) were detected as contaminants and removed from the dataset.


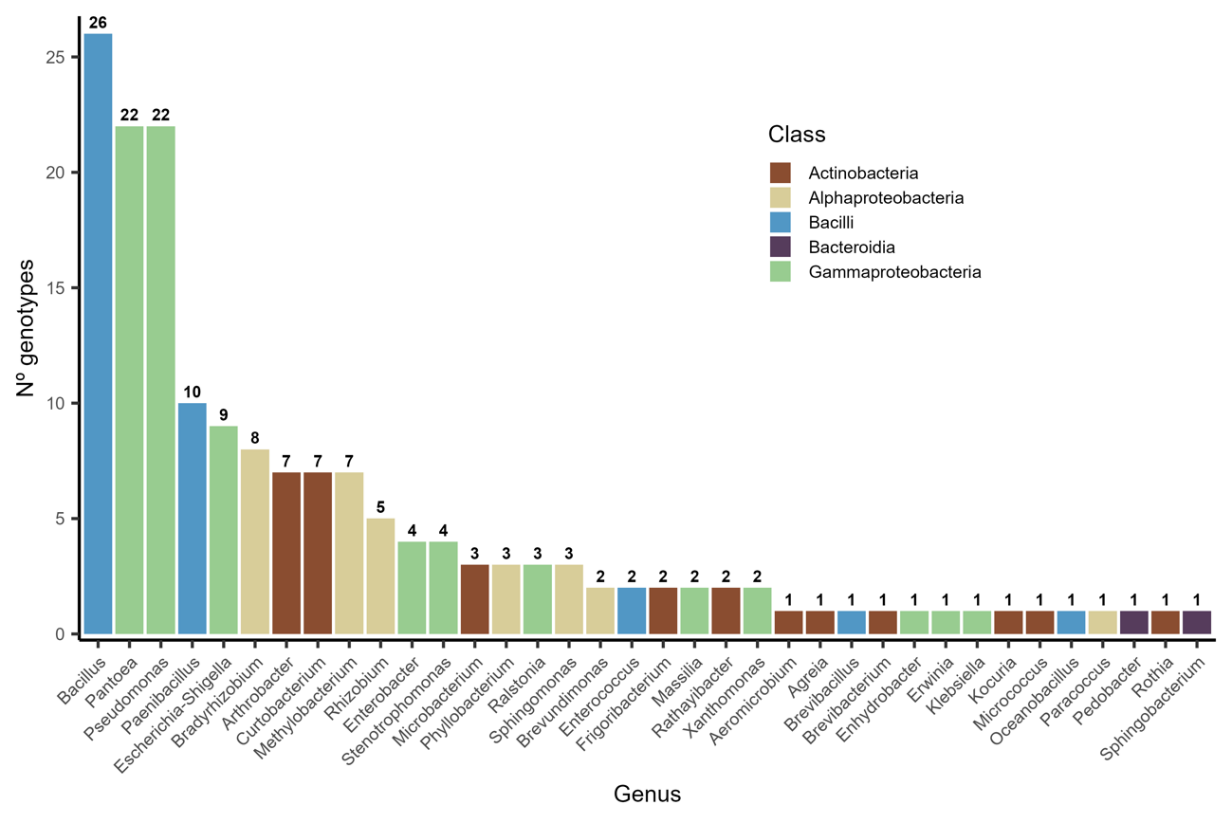
**Figure S3** Prevalence of isolates across genotypes at genus level. Colors indicate the taxonomical classes.


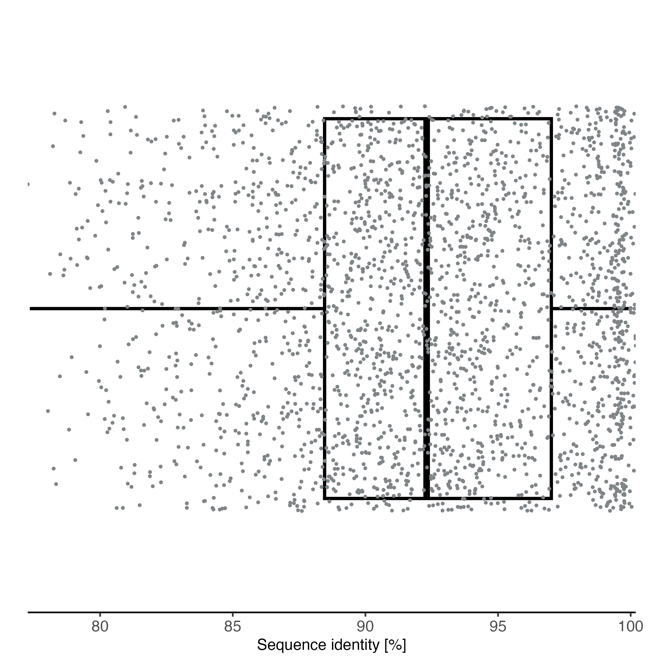


**Figure S4** Sequence identity distribution of all assignments (n = 148 755) between the community ASVs (query) and cultured ASVs (database). Identity percentages were between 77.35% and 100% with a median of 92.31%, and Q1 and Q3 88.46% and 97%, respectively.**
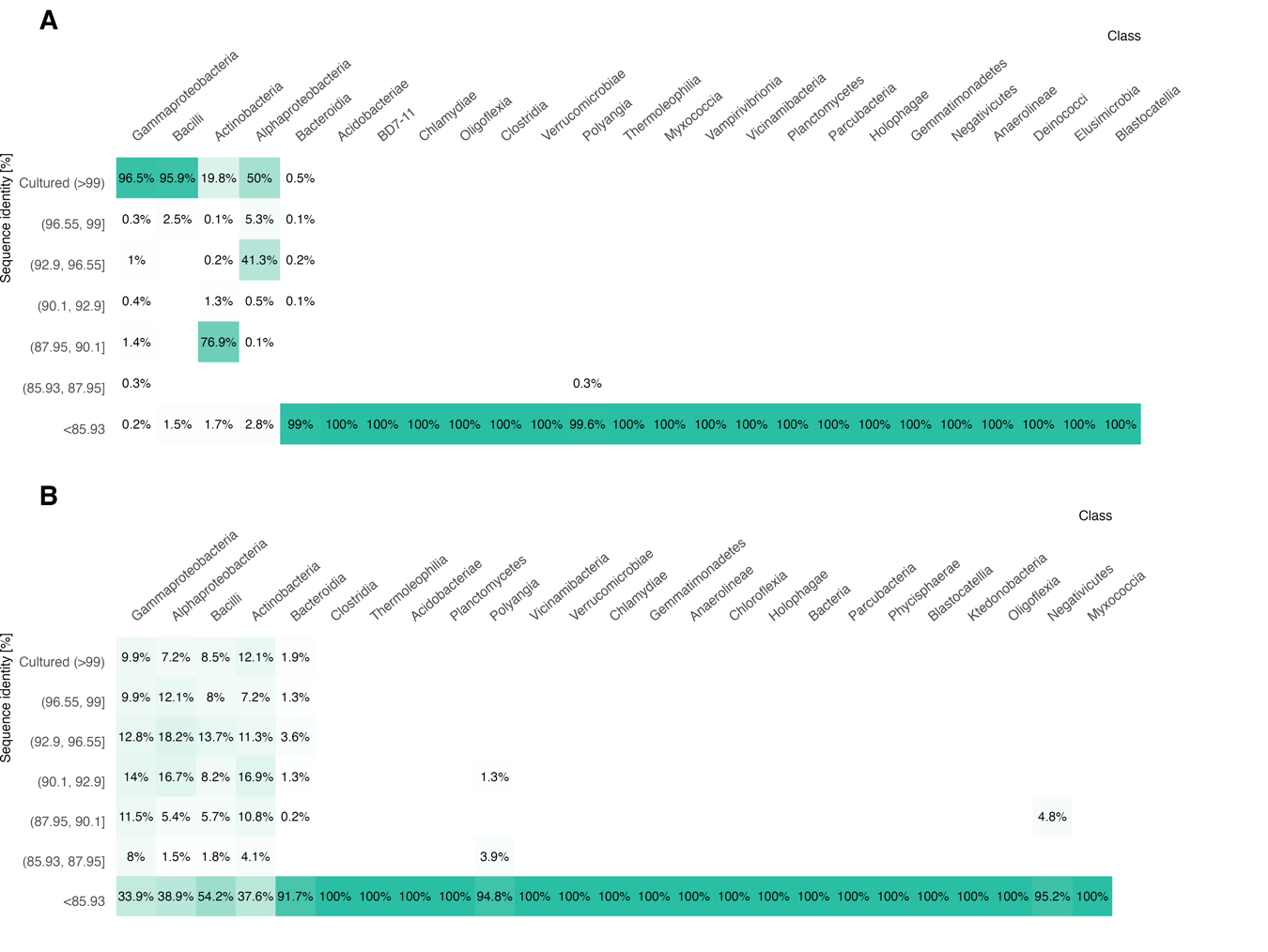
Figure S5** Percentage of the mean relative abundance (**A)** and diversity (**B**) within the different bacterial classes covered by the ASVs matching the cultured taxa at different sequence identities.

**
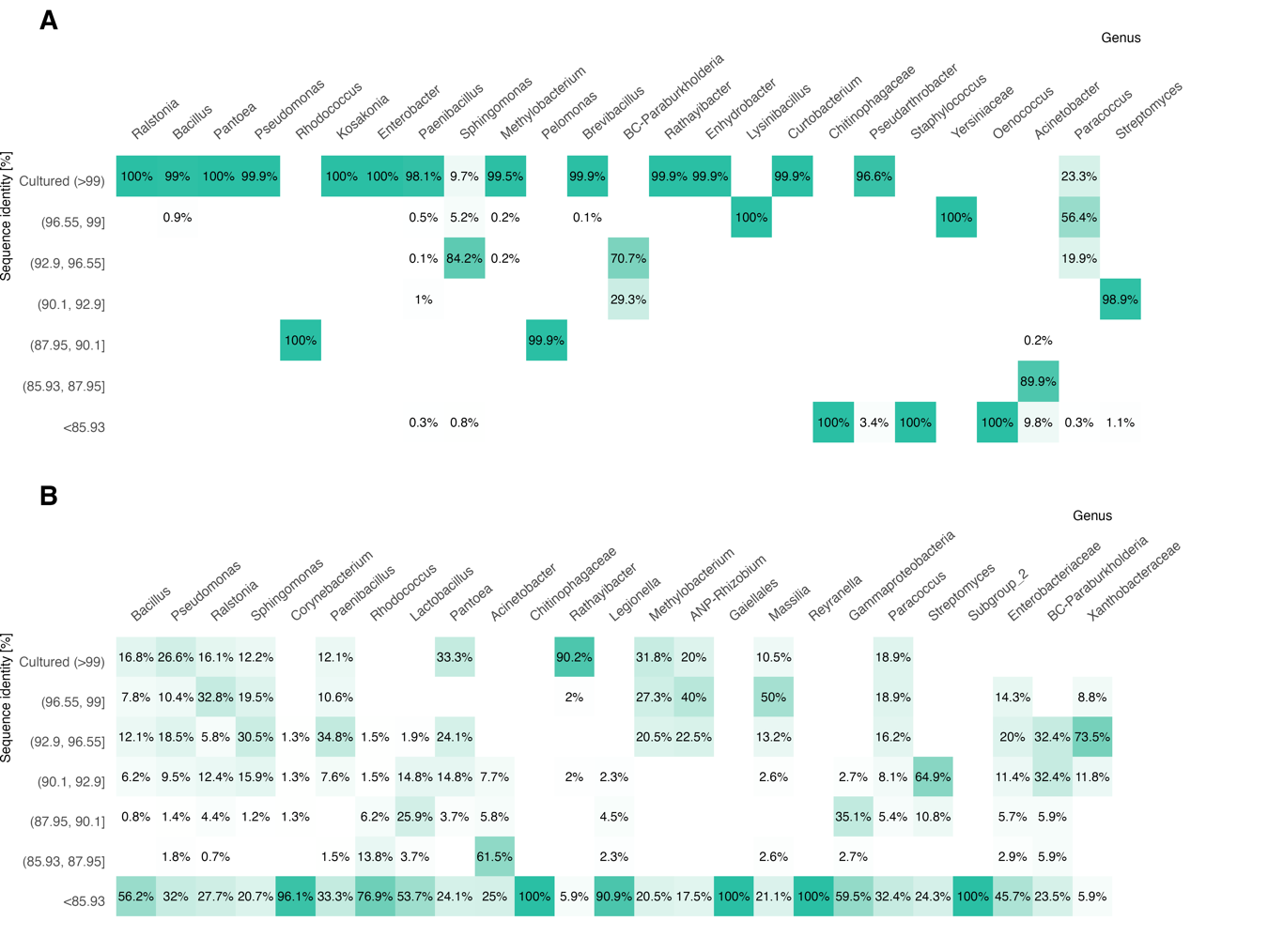
Figure S6** Percentage of the mean relative abundance (**A**) and diversity (**B**) within the different bacterial genera covered by the ASVs matching the cultured taxa at different sequence identities.

**
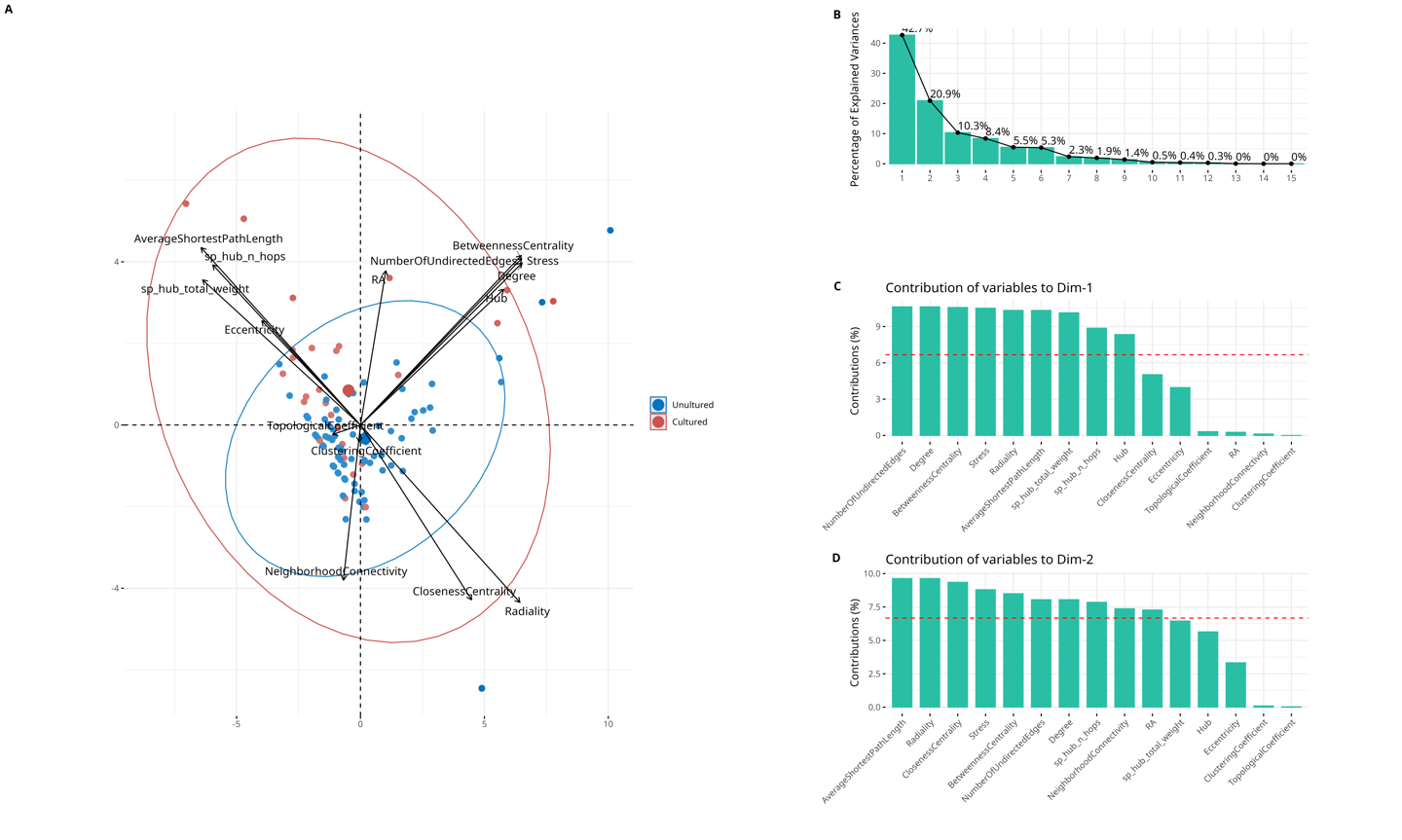
Figure S7** Variance of the cultured and uncultured taxa in the co-occurrence network. (**A)** Principal component analysis (PCA) biplot describing the ASV distribution and the loading vectors of the different experimental variables. (**B)** Percentage of explained variances in the different dimensions. (**C)** Contribution of the experimental variables to the first dimension. (**D)** Contribution of the experimental variables to the second dimension.

Dim-2

Dim-1

42.7%

**
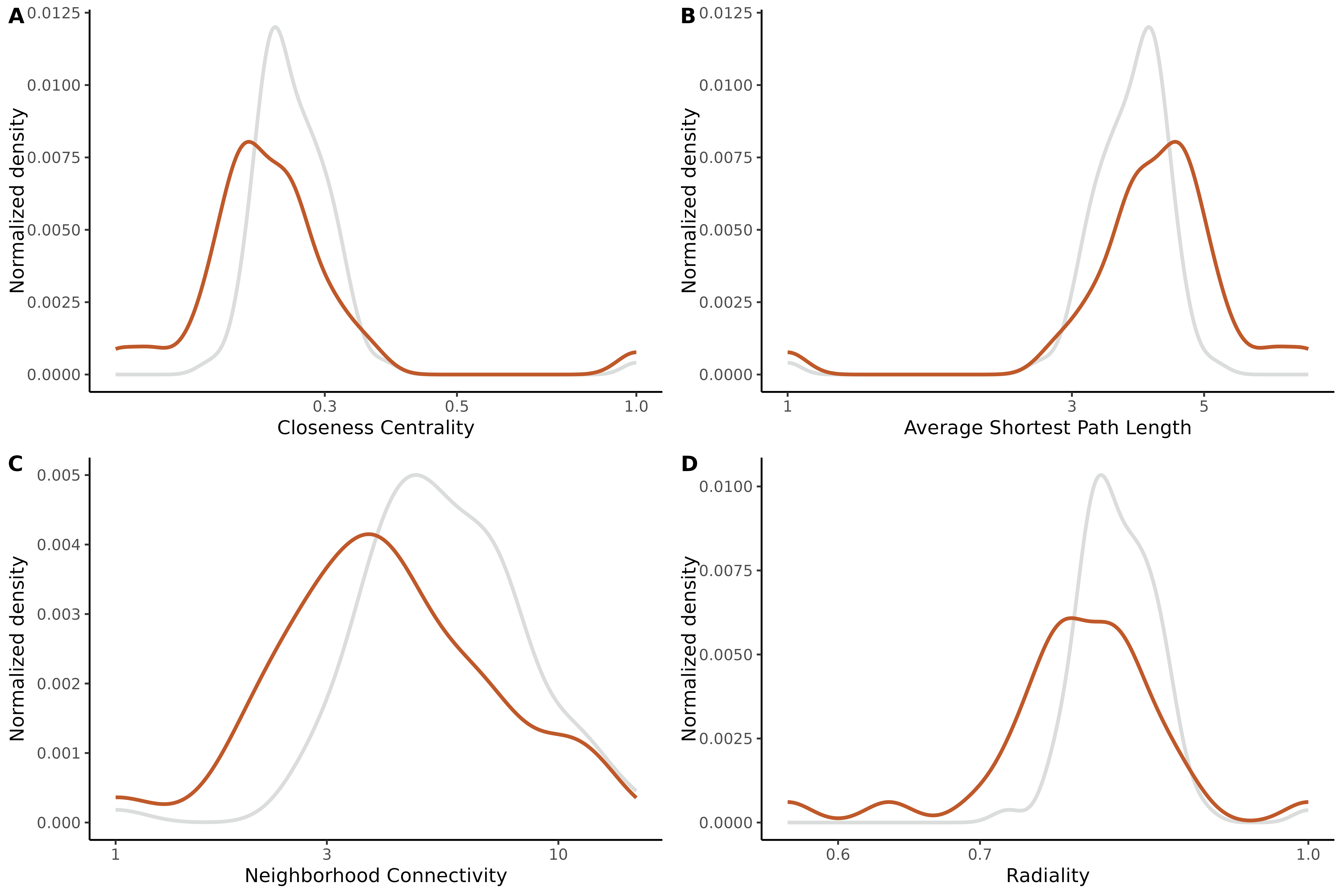
Figure S8** ASVs distribution according to different centrality measures in the cultured and uncultured fractions of the network. Closeness centrality (**A)**, Average shortest path length (**B)**, Neighborhood connectivity (**C)**, and Radiality (**D)**.

**Table S1** Detailed information on the *Cannabis* genotypes used in this study and in Lobato *et al*., (2024). The color code refers to genotypes only investigated in this study (red), only in Lobato *et al*., 2024 (blue) or in both studies (purple).

| **Provider** | **Study / Code** | **Genotype** | **Origin** | **Accession** | **Species** | **Nº Isolations** | **Total Pure Cultures** |
| --- | --- | --- | --- | --- | --- | --- | --- |
| CRI | C65 | Beniko | Poland | 1500003 | *C. sativa ssp. sativa var. sativa* | 1 | 20 |
|  | C64 | Bialobrzeskie | Poland | 1500002 | *C. sativa ssp. sativa var. sativa* | 1 | 27 |
|  | C66 | Fedora | France | 1500011 | *C. sativa ssp. sativa var. sativa* | 1 | 24 |
|  | C67 | Finola | Finland | 1500019 | *C. sativa ssp. sativa var. sativa* | 2 | 50 |
| Green House Seeds Co. | C77 | Arjan's Haze #3 | NA | GHSC052 | *C. sativa ssp. indica var. indica* | - | - |
|  | C76 | The Church CBD | NA | GHSC005 | *C. sativa ssp. indica var. indica* | - | - |
| IPK | C54 | Bredemann P | Germany | CAN 62 | *C. sativa ssp. sativa* | - | - |
|  | C28 | RV Romania 94: 265 | Bulzestii de Sus, Romania | CAN 35 | *C. sativa ssp. sativa* | 1 | 21 |
|  | C26 | RV Romania 94: 226 | Muntele Baisorii, Romania | CAN 33 | *C. sativa ssp. sativa* | 1 | 6 |
|  | C27 | RV Romania 94: 239 | Baia de Aries, Romania | CAN 34 | *C. sativa ssp. sativa* | 1 | 29 |
|  | C31 | RV Romania 94: 115 | Birsana, Romania | CAN 38 | *C. sativa ssp. sativa* | 2 | 25 |
|  | C24 | RV Romania 94: 120 | Strimtura, Romania | CAN 31 | *C. sativa ssp. sativa var. sativa* | 1 | 4 |
|  | C41 | Eletta Campana | Italy | CAN 48 | *C. sativa ssp. sativa var. sativa* | - | - |
|  | C59 | Fasamo | Germany | CAN 67 | *C. sativa ssp. sativa var. sativa* | 1 | 3 |
|  | C44 | Fibridia | Germany | CAN 52 | *C. sativa ssp. sativa var. sativa* | 2 | 26 |
|  | C37 | Fibrimon | Germany | CAN 44 | *C. sativa ssp. sativa var. sativa* | 2 | 36 |
|  | C46 | Fibrimon 21 | Germany | CAN 54 | *C. sativa ssp. sativa var. sativa* | 1 | 28 |
|  | C21 | Forose | NA | CAN 28 | *C. sativa ssp. sativa* | 1 | 21 |
|  | C63 | Gelb | NA | CAN 100 | *C. sativa ssp. sativa var. sativa* | 1 | 7 |
|  | C47 | Havelländer | Germany | CAN 55 | *C. sativa ssp. sativa var. sativa* | - | - |
|  | C36 | Hohenthürmer Gleichzeitig Reifender | Germany | CAN 43 | *C. sativa ssp. sativa var. sativa* | - | - |
|  | C62 | Kompolti | Hungary | CAN 70 | *C. sativa ssp. sativa var. sativa* | 1 | 26 |
|  | C60 | Kompolti PZO | Hungary | CAN 68 | *C. sativa ssp. sativa var. sativa* | 1 | 4 |
|  | C50 | Kongo Hemp | Spain | CAN 58 | *C. sativa ssp. sativa var. sativa* | 1 | 8 |
|  | C38 | Krasnoyarskaya | Russia | CAN 45 | *C. sativa ssp. sativa var. sativa* | 2 | 28 |
|  | C58 | Lovrin 110 | Romania | CAN 66 | *C. sativa ssp. sativa var. sativa* | - | - |
|  | C43 | NA Argentina | Argentina | CAN 51 | *C. sativa ssp. sativa* | - | - |
|  | C34 | NA BG Belgium | NA | CAN 41 | *C. sativa ssp.* | - | - |
|  | C32 | NA BG China | China | CAN 39 | *C. sativa ssp. sativa* | 1 | 16 |
|  | C11 | NA BG Hungary | Hungary | CAN 17 | *C. sativa ssp. sativa* | 1 | 7 |
|  | C33 | NA BG Italy | Carmagnola, Italy | CAN 40 | *C. sativa ssp.* | 1 | 7 |
|  | C15 | NA BG Romania | Laşi, Romania | CAN 21 | *C. sativa ssp. sativa* | - | - |
|  | C30 | NA France | France | CAN 37 | *C. sativa ssp. sativa* | 1 | 29 |
|  | C29 | NA Germany | NA | CAN 36 | *C. sativa ssp.sativa* | 1 | 7 |
|  | C20 | NA Vacratot | NA | CAN 27 | *C. sativa ssp.* | 3 | 53 |
|  | C49 | NA Syria | Syria | CAN 57 | *C. sativa ssp.* | 1 | 16 |
|  | C56 | NA Turkey | Burdur, Turkey | CAN 64 | *C. sativa ssp. sativa var. sativa* | 1 | 9 |
|  | C19 | Turkey NS | Turkey | CAN 26 | *C. sativa ssp.* | 1 | 24 |
|  | C55 | Ramo | Germany | CAN 63 | *C. sativa ssp. sativa var. sativa* | 2 | 10 |
|  | C16 | RV Georgia 86 | Shatili, Georgia | CAN 22 | *C. sativa ssp. sativa var. spontanea* | 1 | 9 |
|  | C18 | RV Italy 84 | Apúlia, Italy | CAN 24 | *C. sativa ssp. sativa* | 2 | 11 |
|  | C13 | RV Italy 88 | Cosenza, Italy | CAN 19 | *C. sativa ssp. sativa* | 1 | 13 |
|  | C17 | RV Korea 87 | North Hwanghae, North Korea | CAN 23 | *C. sativa ssp. sativa var. sativa* | 3 | 25 |
|  | C14 | RV Korea 85 | Kosong, North Korea | CAN 20 | *C. sativa ssp. sativa* | 1 | 16 |
|  | C10 | RV Slovakia 77 | Uličské Krivé, Slovakia | CAN 16 | *C. sativa ssp. sativa* | 1 | 36 |
|  | C52 | Schurig | Germany | CAN 60 | *C. sativa ssp. sativa* | 2 | 19 |
|  | C71 | Superfibra | NA | CAN 49 | *C. sativa ssp.* | 1 | 1 |
| Hanfama GmbH | C08 | Carmagnola | Italy | NA | *C. sativa ssp. sativa var. sativa* | 2 | 62 |
|  | C73 | Félina 32 | France | NA | *C. sativa ssp. sativa var. sativa* | 1 | 20 |
|  | C72 | Férimon | France | NA | *C. sativa ssp. sativa var. sativa* | 1 | 41 |
|  | C74 | Futura 75 | France | NA | *C. sativa ssp. sativa var. sativa* | 1 | 10 |
|  | C75 | Orion 33 | France | NA | *C. sativa ssp. sativa var. sativa* | 1 | 23 |
|  | C09 | Santhica 27 | France | NA | *C. sativa ssp. sativa var. sativa* | 3 | 123 |
| Hanfland GmbH | C01 | Fedora 17 | France | NA | *C. sativa ssp. sativa var. sativa* | 2 | 31 |
| KFU Botanical Garden | C02 | Silvana x Manitoba Poison | Romania/South Africa | NA | *C. sativa ssp. sativa var. sativa x ssp. indica var. indica* | 1 | 12 |
| LVMI Silava | C70 | Purini | Latvia | LVA02775 | *C. sativa ssp. sativa* | - | - |
|  | C69 | Vietejas | Latvia | LVA02701 | *C. sativa ssp. sativa* | 1 | 29 |
| Plant Breeding and Acclimatization Institute | C83 | Bernburskie | Poland | 501000 | *C. sativa ssp. sativa* | 1 | 15 |
|  | C04 | Dolnoslaskie | Poland | 166502 | *C. sativa ssp. sativa* | 1 | 12 |
|  | C84 | Futura 77 | France | 501002 | *C. sativa ssp. sativa* | 1 | 11 |
|  | C07 | Lizap 12 119 | Poland | 503007 | *C. sativa ssp. sativa* | 3 | 25 |
|  | C06 | NA Poland | Poland | 166504 | *C. sativa ssp. sativa* | 1 | 17 |
| Sensi Seeds | C87 | Afghani #1 | Afghanistan | 1500010 | *C. sativa ssp. indica* | 1 | 7 |
|  | C86 | Hindu Kush | Afghanistan | 1500001 | *C. sativa ssp. indica* |  |  |
|  | C91 | Master Kush | Afghanistan | 1510002 | *C. sativa ssp. indica var. indica* | - | - |
|  | C90 | Mexican Sativa | NA | 1500038 | *C. sativa ssp. indica var. indica* | 1 | 18 |
|  | C89 | Ruderalis Indica | NA | 1500034 | *C. sativa ssp. indica var. indica x ssp. sativa var. spontanea* | 1 | 13 |
|  | C88 | Skunk #1 | NA | 1500024 | *C. sativa ssp. indica var. indica* | - | - |

**Table S2** Detailed information on the media used for bacterial isolation.

| **Media** | **Composition** | **Amount / 1L dH_2_O** |
| --- | --- | --- |
| NA | Nutrient Broth II (Sifin) | 15 g |
|  | Agar (Roth) | 18 g |
|  | Nystatin (Roth) — 25 mg/mL pure Ethyl alcohol | 1 mL |
| LB | Luria/Miller Broth (Roth) | 20 g |
|  | Agar (Roth) | 18 g |
|  | Nystatin (Roth) — 25 mg/mL pure Ethyl alcohol | 1 mL |
| TSA | Tryptic Soy Broth (Roth) | 30 g |
|  | Agar (Roth) | 18 g |
|  | Nystatin (Roth) — 25 mg/mL pure Ethyl alcohol | 1 mL |
| R2A | Reasoner's 2A agar (Roth) | 18 g |
|  | Nystatin (Roth) — 25 mg/mL pure Ethyl alcohol | 1 mL |
| Kings’s B | Proteose peptone (Roth) | 2 g |
|  | K_2_HPO_4,_ (Roth) | 0.15 g |
|  | MgSO_4_.xH_2_O (Roth) | 0.15 g |
|  | Glycerol >99.5 % (Roth) | 1 g |
|  | Agar (Roth) | 15 g |
|  | Nystatin (Roth) — 25 mg/mL pure Ethyl alcohol | 1 mL |
| PDA | Potato Dextrose Agar (Roth) | 26.5 g |
|  | Nystatin (Roth) — 25 mg/mL pure Ethyl alcohol | 1 mL |
| 869 (1/10) | Tryptone/peptone (Roth) | 1 g |
|  | Yeast extract (Roth) | 0.5 g |
|  | NaCl (Roth) | 0.5 g |
|  | D-Glucose (Roth) | 0.1 g |
|  | CaCl_2_.2H_2_O (Roth) | 0.04 g |
|  | *Cannabis* extracts — Fedora 17 | 10 mL |
|  | Agar (Roth) | 18 g |
|  | Nystatin (Roth) — 25 mg/mL pure Ethyl alcohol | 1 mL |
| M9 | M9 minimal medium (Sigma-Aldrich) | 11.28 g |
|  | *Cannabis* extracts — Fedora 17 | 10 mL |
|  | Agar (Roth) | 18 g |
|  | Nystatin (Roth) — 25 mg/mL pure Ethyl alcohol | 1 mL |

**Table S3** Megablast alignment output from Galaxy between the community ASVs (query) and cultured ASVs (database) resulted in 148.755 assignments. The table describes the coverage length, number of mismatches, e-val and bitscore values for each assignment.

- See separate file **Table S3.csv**

**Table S4** Differences in the mean relative abundance distribution assessed through Mann-Whitney-U-Test between **A** uncultured ASVs with different sequence identities to the cultured isolates and phylogenetically distant ASVs (< 85.93% sequence similarity to the isolates), and **B** uncultured ASVs and cultured ASVs. Significant differences (α = 0.05) are marked (*).

| **A** |  | **W** | **p-value** |
| --- | --- | --- | --- |
| Uncultured (96.55, 99) | Uncultured (< 85.93) | 665021 | < 2.2e-16* |
| Uncultured (92.9, 96.55) | Uncultured (< 85.93) | 766269 | 0.00019* |
| Uncultured (90.1, 92.9) | Uncultured (< 85.93) | 722696 | 0.05558 |
| Uncultured (87.95, 90.1) | Uncultured (< 85.93) | 448283 | 0.1724 |
| Uncultured (85.93, 87.95) | Uncultured (< 85.93) | 258748 | 0.001163* |

| **B** |  | **W** | **p-value** |
| --- | --- | --- | --- |
| Uncultured (96.55, 99) | Cultured (>99) | 45997 | 3.279e-05***** |
| Uncultured (92.9, 96.55) | Cultured (>99) | 46674 | < 2.2e-16***** |
| Uncultured (90.1, 92.9) | Cultured (>99) | 43744 | < 2.2e-16***** |
| Uncultured (87.95, 90.1) | Cultured (>99) | 26983 | < 2.2e-16***** |
| Uncultured (85.93, 87.95) | Cultured (>99) | 16891 | 8.752e-12***** |
| Uncultured (< 85.93) | Cultured (>99) | 705211 | < 2.2e-16* |
